# Supplementary material for: Generating realistic neurophysiological time series with denoising diffusion probabilistic models
Source: Patterns (N Y). 2024 Aug 29;5(9):101047. doi: 10.1016/j.patter.2024.101047 (PMC11573898; doi:10.1016/j.patter.2024.101047)
Supplement: Document S1. Supplemental experimental procedures, Figures S1‒S12, and Tables S1 and S2 [file mmc1.pdf]

Patterns, Volume *5*

Supplemental information

Generating realistic neurophysiological time series with denoising diffusion probabilistic models

Julius Vetter, Jakob H. Macke, and Richard Gao

Supplemental Information

Supplemental Experimental Procedures

Ornstein-Uhlenbeck process as diffusion process

In our experiments, we sometimes use the OU process instead of the classically used white noise as our diffusion process. This substitution is possible because the OU process belongs to the family of Gaussian processes. See Biloš et al.[1] for more details.

The training and sampling schemes are shown in Algorithm 1 and Algorithm 2 and differ slightly from those given in Biloš et al.[1]. Note that the extension to general Gaussian processes has a computational overhead compared to the original training objective. During training, the computation of the Mahalanobis distance scales quadratically with the length of the input. Sampling from a Gaussian process also requires computing a Cholesky decomposition. However, this decomposition only needs to be computed once, and can also be used to compute the Mahalanobis efficiently by solving a lower triangular linear system based on the Cholesky decomposition.

Algorithm 1 Training

$L = \mathrm{Cholesky}(\Sigma)$
repeat
$\mathbf{x}_0 \sim q(\mathbf{x}_0)$
$t \sim \mathrm{Uniform}(\{1, \ldots, T\})$
$\boldsymbol{\epsilon} \sim \mathcal{N}(0, \Sigma)$
Take gradient descent step on
  $\nabla_\theta (\boldsymbol{\epsilon} - \boldsymbol{\epsilon}_\theta(\mathbf{x}_t, t))^T \Sigma^{-1} (\boldsymbol{\epsilon} - \boldsymbol{\epsilon}_\theta(\mathbf{x}_t, t))$
until converged

Algorithm 2 Sampling

$\mathbf{x}_T \sim \mathcal{N}(0, \Sigma)$
for $t = T, \ldots, 1$ **do**
  $z \sim \mathcal{N}(0, \Sigma)$ if $t > 1$, else $z = 0$
  $\mathbf{x}_{t-1} = \frac{1}{\sqrt{\alpha_t}}(\mathbf{x}_t - \frac{1-\alpha_t}{\sqrt{1-\bar{\alpha}_t}} \boldsymbol{\epsilon}_\theta(\mathbf{x}_t, t)) + \sigma_t z$
end for
return $\mathbf{x}_0$

As described in the main text, the computational requirements scale more favorably for the special case of the OU process. Its formulation as a stochastic differential equation allows sampling in linear time. Furthermore, the OU process has a tridiagonal precision matrix, which allows computing the training objective in linear time as well.

Additional results and details

Here we provide additional results and details. The hyperparameters used for our experiments are given in Table S2.

For completeness, all distributions of PSDs of AJILE12 participant P07 in the main text are given (Fig. S7). Additionally, all distributions of PSDs for two other participants, participant P01 with 94 channels (Fig. S8) and participant P12 with 126 channels (Fig. S9) are provided. In both cases, the median spectra as well as 10 % and 90 % percentiles match well. Recall that the neural decoding performance on data imputed by our model did not improve over the mean imputation baseline for participant P01. Nevertheless, the overall spectra of the real and imputed data match. This means that while the model was able to capture the overall distribution of time series, it likely failed to learn the class-conditional mapping between observed and missing channels. The average correlations between ground truth and imputations are positive for all 12 participants. With increasing levels of dropout, they drop towards zero (Table S1). The relative improvements of the DDPM-based imputations over the mean imputation baseline are on average better than the ones obtained by the deterministic CNNAE model (Talukder et al.[2], Table S1). We also provide an example of a deterministic, neural network-based imputation (Fig. S5).

As an additional experiment with high channel count, we generated time series from the awake condition of the macaque ECoG data on all 128 recorded channels. The distribution of real and generated PSDs closely match across all channels, but our model sometimes overestimates the power in the low-frequency region (Fig. S6).

Distances between real and generated data For all datasets, we generate the same number of samples as were used to train the corresponding models. We then compute all nearest-neighbor distances within the training data and from the generated samples to the training samples. Two distance measures are used, the L_1 distance in

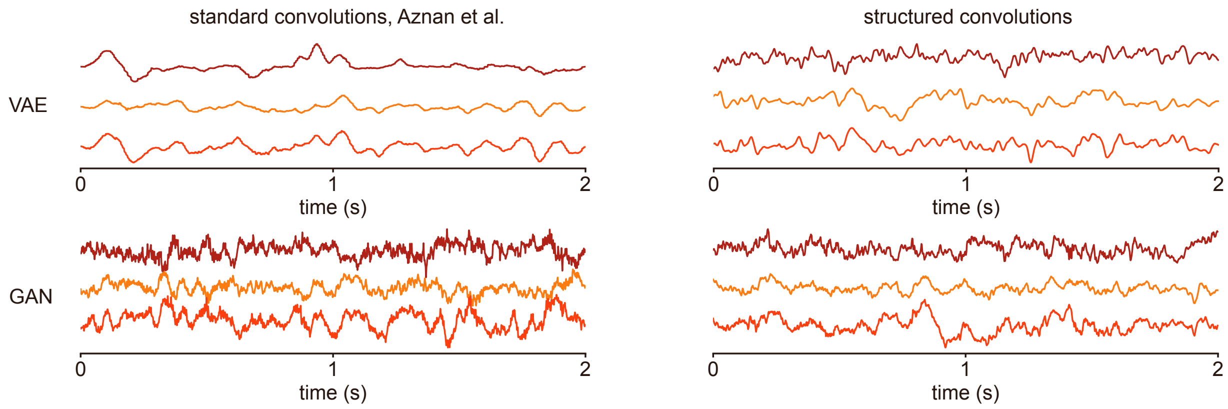

Figure S1: **Samples from GANs and VAEs with architectures based on standard and structured convolutions** The encoder/decoder and generator/discriminator architectures based on structured convolutions produce better-looking samples.

the time domain (which is small if and only if two time series are nearly exact copies of each other) and the Fourier-Wasserstein distance[3]. The Fourier-Wasserstein distance, a pseudo-metric that operates in the spectral domain, is based on normalized PSDs and will be small if, for example, two time series are scaled or translated versions of each other.

We then compare the distribution of nearest neighbor distances between the real and generated data with the distribution of distances within the training data (Fig. S4). Crucially, the minimum nearest-neighbor distance between and within the real and generated time series is away from zero for both the L_1 and the Fourier-Wasserstein distance and similar to the distribution of distances within. For a heavily overfitted DDPM, we would expect large parts of the distribution of nearest-neighbor distances between the real and generated data to be substantially smaller.

OU process versus white noise Our use of the OU process provides a modification that can further improve the quality of generated samples in some cases. Here, we provide an analysis for the AJILE12 dataset. We retrain the models using the standard independent Gaussian process (white noise). All other training and network hyperparameters are kept fixed.

For all AJILE12 participants (Fig. S10, Fig. S11, Fig. S12 for AJILE12 participants P01, P07, P12, respectively), the median power-spectra are worse using the white noise instead of the OU process, especially in the low-power, high-frequency region, which is overestimated when white noise is used.

However, for the rat LFP, the awake/anesthetized macaque ECoG data, and the BCI EEG data, there was no substantial advantage to using the OU process over white noise.

VAE and GAN baseline Here, we use the unconditional rat LFP generation experiment to establish a baseline using other types of generative models. We train VAEs and GANs using the architectures from Aznan et al.[4] as well as our own encoder/decoder and generator/discriminator architectures based on structured convolutions. The convolution hyperparameters were informed by those used in DDPM, but further tuned for the generative model at hand.

We find that the architectures used in Aznan et al.[4] are not sufficient to faithfully capture the statistics of the LFP recordings. Our architectures based on structured convolutions produce more realistic samples for both VAE and GAN (Fig. S1). However, and as we discuss below, they still fall short of the performance achieved by the DDPM:

The VAE with the encoder/decoder based on structured convolutions produces realistic but too smooth LFPs (Fig. S2A). The marginals between real and generated recordings closely match (Fig. S2B). At low frequencies, the VAE-generated samples capture the spectrum of the recordings well. At higher frequencies, however, their power drops off too quickly (Fig. S2C). As a result, the generated samples appear smooth or blurred. This is a common problem with VAEs[5,6]. Because of their smoothness, the samples do not have the correct phase-amplitude coupling (Fig. S2D).

The GAN with the generator/discriminator based on structured convolutions is able to produce realistic looking LFP recordings (Fig. S2A) whose power spectra and marginals match (Fig. S2B and C). Furthermore, we observe a non-trivial phase-amplitude coupling in the generated samples (Fig. S2D). Overall, however, the quality of the generated samples is inferior to that of the DDPM: The median spectra show strong oscillations at higher frequencies, and the couplings only approximately match those of the real data. Furthermore, we found the training of the GAN

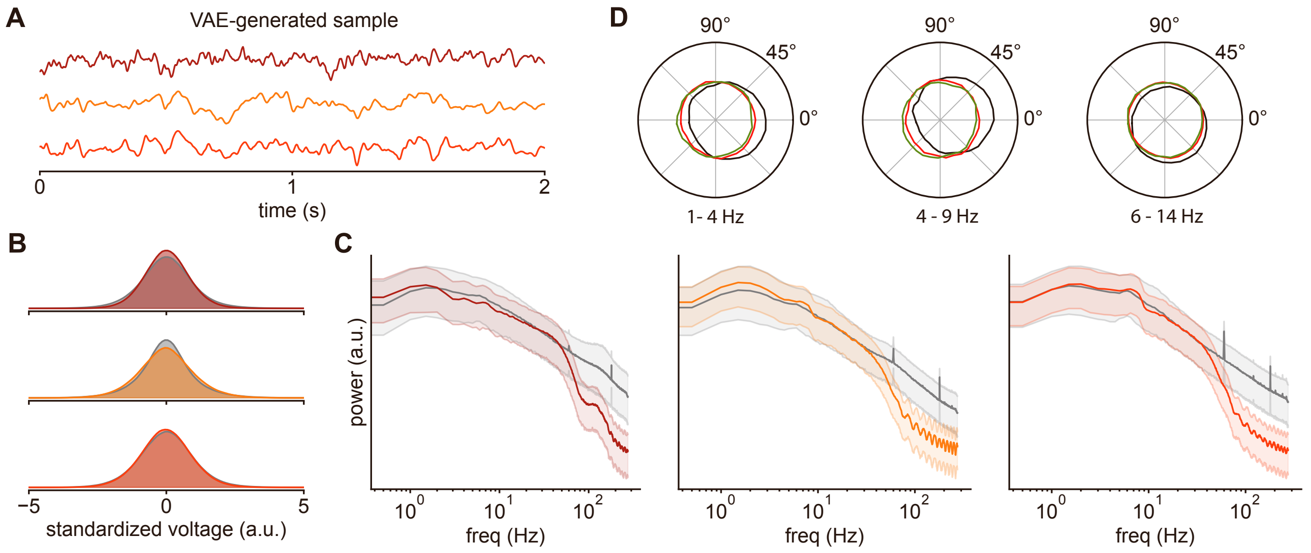

Figure S2: **VAE with structured convolutions for rat cortical-hippocampal LFP. (A)** Example of time series generated by the VAE. **(B)** Marginal distributions over the standardized voltage of each channel for both real and generated data. **(C)** Median and 10 %/90 % percentiles of real and generated power spectra for each channel. **(D)** Phase-amplitude and phase-count coupling of CA1 ripples for different driver frequencies in the mPFC for real, surrogate, and VAE-generated data.

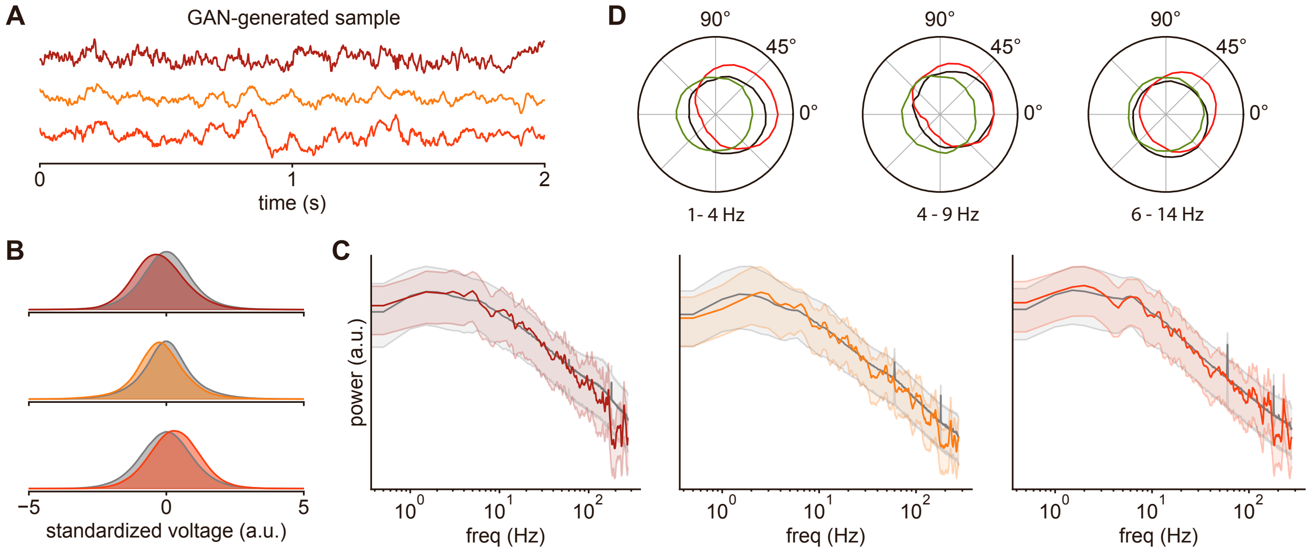

Figure S3: **GAN with structured convolutions for rat cortical-hippocampal LFP. (A)** Example of time series generated by the GAN. **(B)** Marginal distributions over the standardized voltage of each channel for both real and generated data. **(C)** Median and 10 %/90 % percentiles of real and generated power spectra for each channel. **(D)** Phase-amplitude and phase-count coupling of CA1 ripples for different driver frequencies in the mPFC for real, surrogate, and GAN-generated data.

to be relatively unstable[7,8]. Different seeds can lead to very different results, even if the chosen hyperparameters are kept fixed.

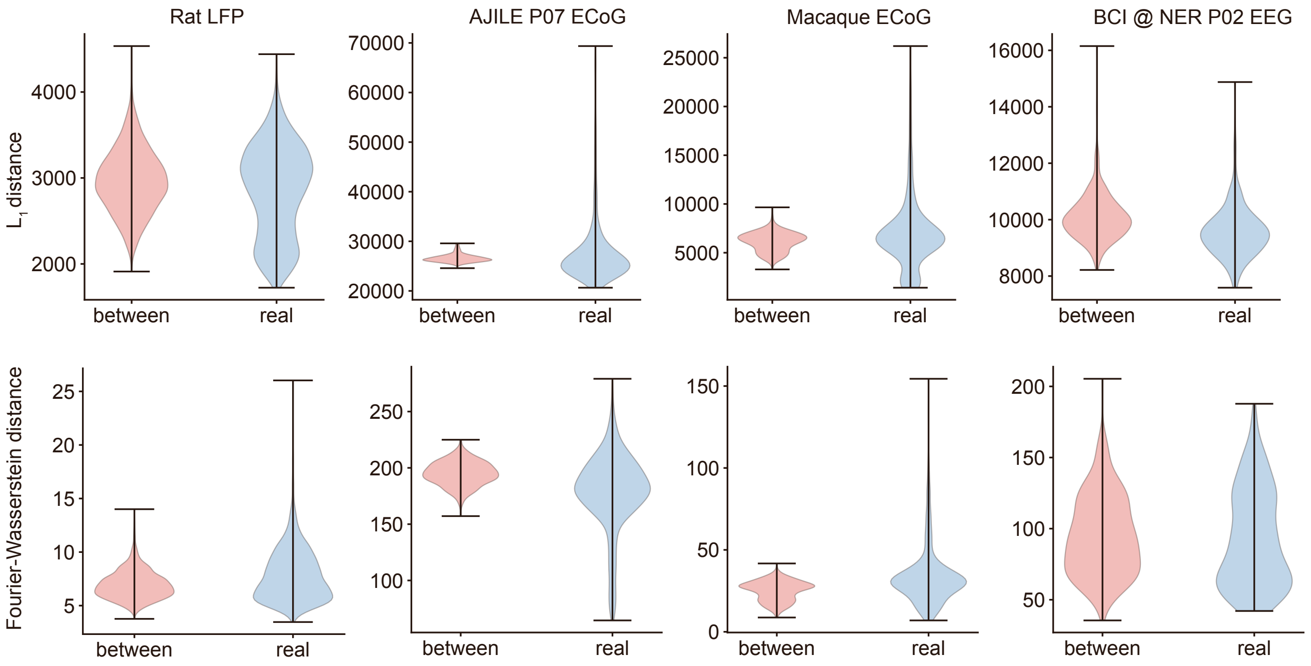

Figure S4: **Distribution of nearest-neighbor distances.** Nearest neighbors were computed from all generated to all real training time series, as well as within all training time series. The whiskers show the minimum and maximum distance (L_1 or the Fourier-Wasserstein distance). The minimum nearest-neighbor distance between real and generated time series is away from zero and similar to or larger than the minimum distance within the set of training time series.

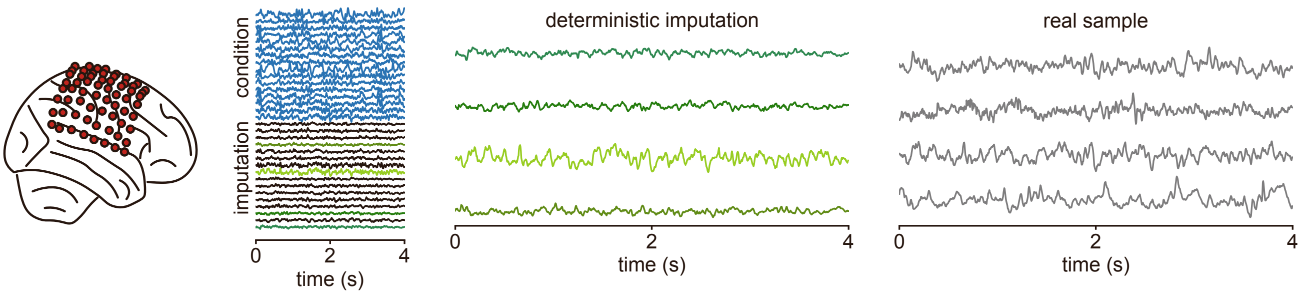

Figure S5: **Example of deterministic neural network-based imputation for the AJILE12 dataset.** The imputation shown is for participant P07. The deterministic imputation appears too flat when compared to the real sample.

Table S1: **Correlations and decoding accuracies for AJILE12 experiment.** Average correlation between imputed and ground truth channels for all test time series across 5 different dropout seeds for diffusion and neural network based imputations. Number in brackets is standard deviation over different dropout seeds. Relative improvement of decoding accuracy over mean imputation in percentage points (pp) when using either DDPM, neural-network or CNNAE imputations[2]. The average and standard deviation over 5 different dropout seeds are shown. First three rows contain the average across all participants.

Pt.	Drop.	Correlation (DDPM)	Correlation (deterministic)	Imp. in pp (DDPM)	Imp. in pp (deterministic)	Imp. in pp Talukder et al.[2]
AVG.	50 %	0.27	0.50	5.9	3.6	3.2
	70 %	0.21	0.45	7.4	6.1	4.6
	90 %	0.10	0.32	4.0	2.5	2.7
P01	50 %	0.37 (0.025)	0.54 (0.026)	−4.6 (5.69)	−10.8 (13.37)	0.2 (0.34)
	70 %	0.28 (0.011)	0.47 (0.013)	0.1 (0.07)	0.0 (0.0)	−0.2 (0.44)
	90 %	0.12 (0.021)	0.32 (0.034)	0.0 (0.04)	0.0 (0.04)	−0.2 (0.39)
P02	50 %	0.42 (0.024)	0.61 (0.020)	2.3 (2.99)	3.1 (2.86)	−1.3 (1.27)
	70 %	0.37 (0.014)	0.58 (0.009)	3.9 (4.55)	5.7 (2.82)	0.5 (2.17)
	90 %	0.18 (0.062)	0.43 (0.049)	4.1 (5.28)	3.9 (2.40)	0.3 (1.08)
P03	50 %	0.30 (0.016)	0.54 (0.015)	8.8 (4.58)	3.9 (1.43)	4.3 (2.66)
	70 %	0.24 (0.006)	0.49 (0.011)	13.7 (3.13)	6.2 (3.69)	4.8 (2.91)
	90 %	0.10 (0.027)	0.32 (0.038)	4.8 (4.07)	0.6 (0.69)	3.6 (4.26)
P04	50 %	0.19 (0.019)	0.38 (0.012)	2.8 (1.88)	6.1 (2.74)	5.0 (2.40)
	70 %	0.15 (0.012)	0.36 (0.022)	4.4 (1.92)	8.8 (1.41)	6.7 (1.81)
	90 %	0.06 (0.012)	0.23 (0.026)	1.0 (1.12)	1.9 (1.93)	1.5 (1.49)
P05	50 %	0.15 (0.007)	0.41 (0.007)	6.8 (2.04)	3.7 (1.02)	2.5 (1.30)
	70 %	0.12 (0.005)	0.37 (0.009)	7.2 (2.24)	3.6 (0.97)	3.1 (0.28)
	90 %	0.05 (0.004)	0.26 (0.011)	2.4 (1.81)	1.2 (0.56)	1.8 (0.88)
P06	50 %	0.11 (0.014)	0.37 (0.020)	0.1 (6.27)	0.6 (3.02)	2.4 (2.37)
	70 %	0.08 (0.009)	0.34 (0.005)	1.8 (3.76)	3.6 (1.22)	1.8 (1.56)
	90 %	0.03 (0.004)	0.23 (0.006)	0.2 (1.11)	−2.2 (3.01)	2.8 (1.33)
P07	50 %	0.35 (0.018)	0.59 (0.015)	14.0 (3.70)	10.6 (3.51)	9.5 (4.77)
	70 %	0.27 (0.025)	0.53 (0.016)	20.7 (1.37)	16.2 (2.68)	13.5 (3.05)
	90 %	0.13 (0.031)	0.38 (0.037)	14.5 (5.23)	7.6 (5.37)	6.7 (1.63)
P08	50 %	0.17 (0.015)	0.43 (0.018)	4.4 (4.37)	2.4 (3.51)	0.6 (2.42)
	70 %	0.13 (0.018)	0.39 (0.021)	2.5 (4.52)	5.7 (1.79)	3.0 (6.00)
	90 %	0.04 (0.007)	0.24 (0.019)	2.8 (1.38)	6.6 (1.03)	1.8 (0.47)
P09	50 %	0.27 (0.021)	0.52 (0.010)	6.5 (2.09)	3.2 (1.51)	−5.7 (1.84)
	70 %	0.21 (0.010)	0.47 (0.010)	5.9 (3.20)	3.5 (1.08)	0.8 (2.01)
	90 %	0.08 (0.012)	0.32 (0.013)	6.7 (2.39)	2.7 (1.92)	1.0 (1.17)
P10	50 %	0.30 (0.011)	0.53 (0.013)	16.1 (15.37)	8.1 (13.57)	10.2 (2.69)
	70 %	0.23 (0.018)	0.47 (0.022)	9.3 (12.89)	3.0 (9.75)	4.7 (5.57)
	90 %	0.14 (0.008)	0.36 (0.004)	1.6 (1.14)	0.5 (0.28)	0.7 (1.31)
P11	50 %	0.28 (0.014)	0.52 (0.016)	4.5 (4.97)	5.2 (5.04)	7.0 (6.97)
	70 %	0.23 (0.018)	0.47 (0.016)	9.8 (5.04)	8.3 (5.09)	11.8 (5.59)
	90 %	0.12 (0.014)	0.34 (0.018)	6.4 (5.45)	5.3 (3.63)	10.1 (7.89)
P12	50 %	0.28 (0.016)	0.55 (0.018)	8.6 (2.68)	8.0 (0.59)	3.5 (1.65)
	70 %	0.22 (0.011)	0.51 (0.011)	10.0 (2.33)	8.6 (1.57)	4.9 (0.84)
	90 %	0.11 (0.019)	0.37 (0.020)	3.6 (1.77)	1.8 (0.47)	1.7 (1.20)

Table S2: **Hyperparameters used to train models.** For the AJILE dataset, he same hyperparameters were used for all 12 participants, with the exception of the number of training epochs, to account for the large differences in training data size between participants

**Hyperparameter**	**LFP (rat)**	**AJILE12**	**ECoG (macaque)**	**EEG BCI**
In kernel size	32	1	32	65
Out kernel size	32	1	32	65
SC layers	3	3	3	3
SC kernel size	32	53	101	65
SC scales	5	4	1	1
Latent dim. per channel	64	16	32	16
Off-diag. size	64	4	4	8
Noise process	White noise	OU ($\rho = 10$)	White noise	White noise
Diffusion steps T	500	50	500	500
Learning rate	0.0001	0.0005	0.0004	0.0004
Weight decay	0.01	0.01	0.01	0.01
Train batch size	32	32	32	32
Epochs	500	250/500/1000	500	1000

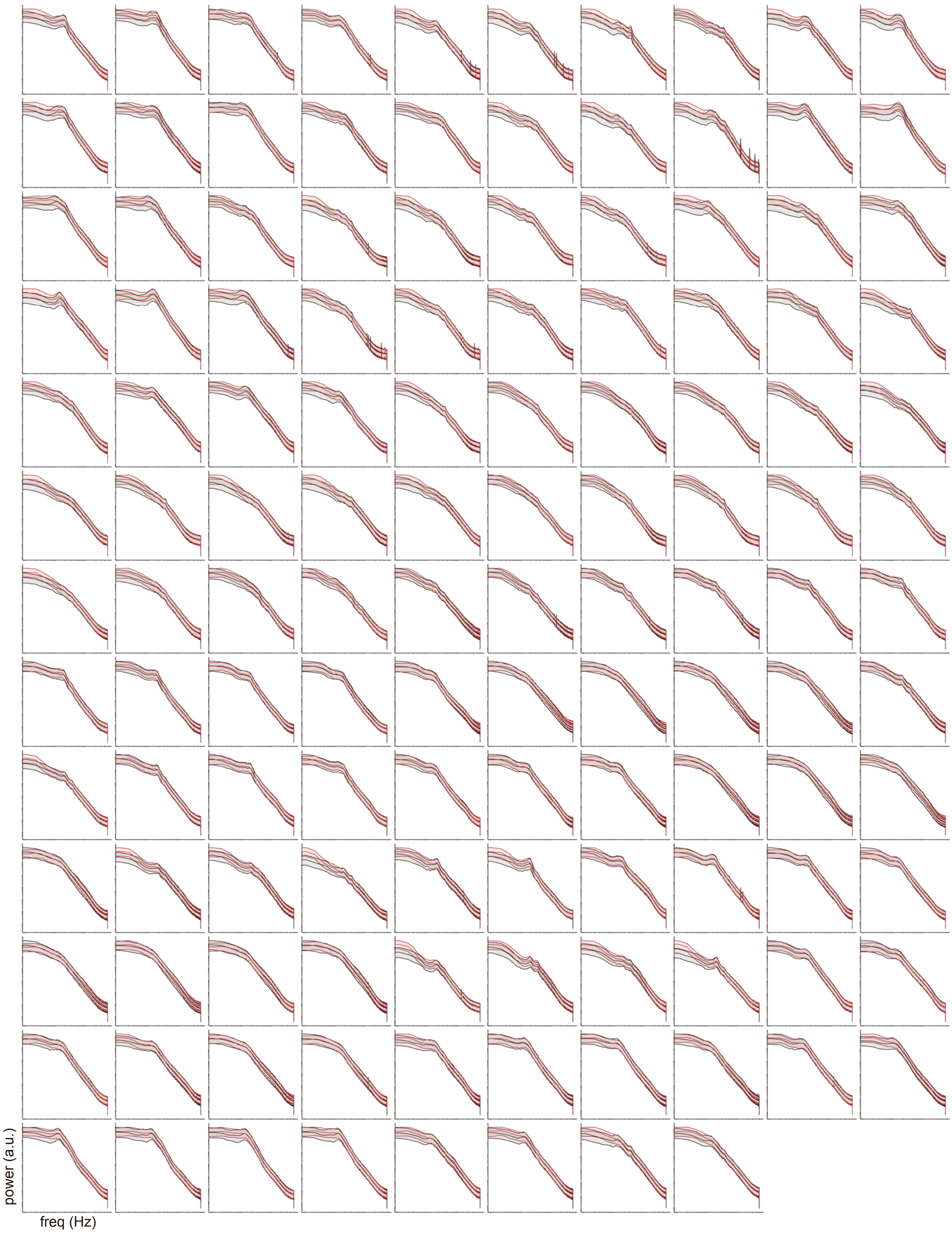

Figure S6: **Full spectra of 128 channel macaque data in awake condition.** Median power as well as 10 %/90 % percentiles are shown.

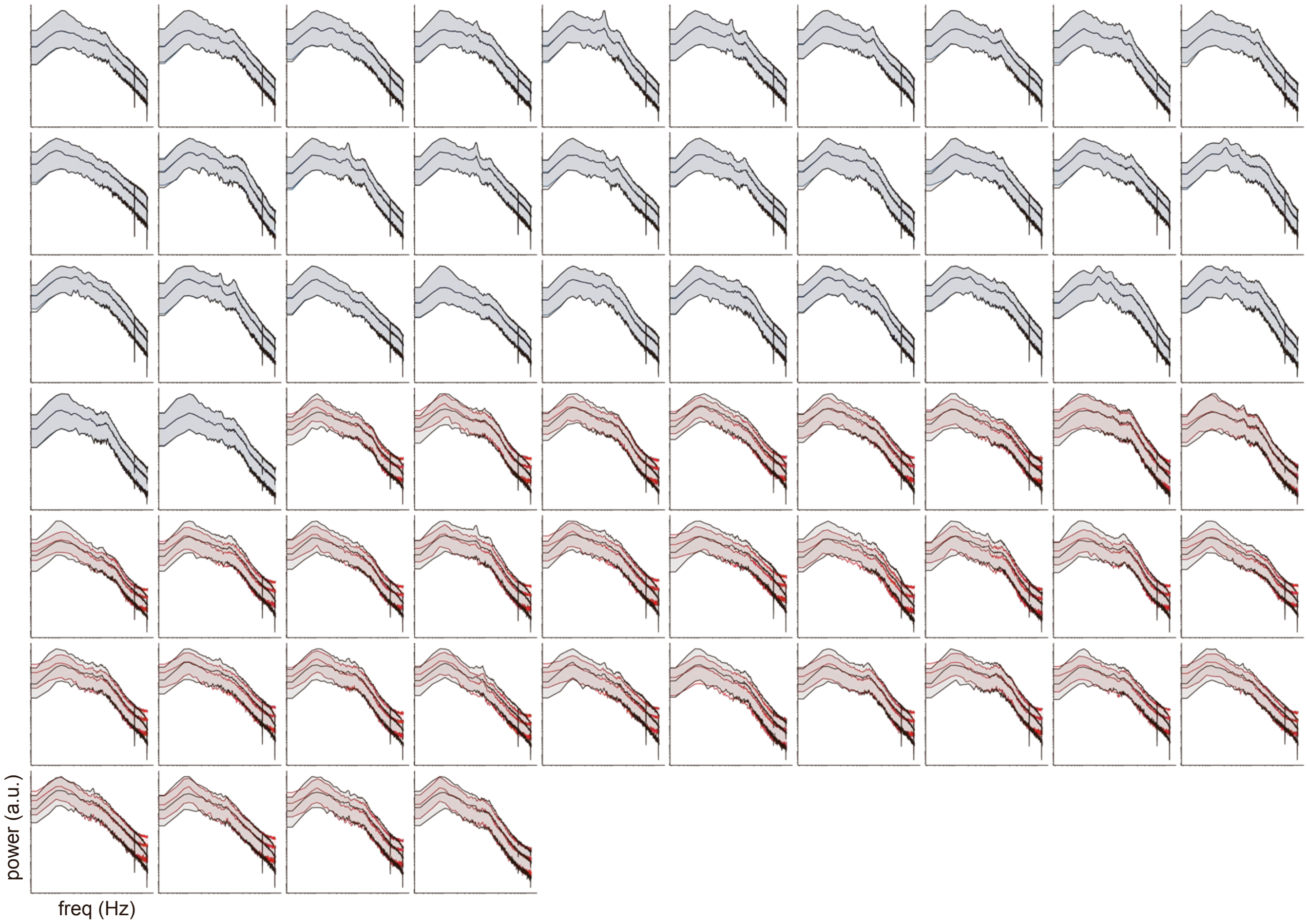

Figure S7: **Full spectra of P07 from the AJILE12 dataset.** The LFP traces consist of 64 channels. Here, we imputed the second half of the channels given the first half for all time series in the evaluation set. Median power as well as 10 %/90 % percentiles are shown.

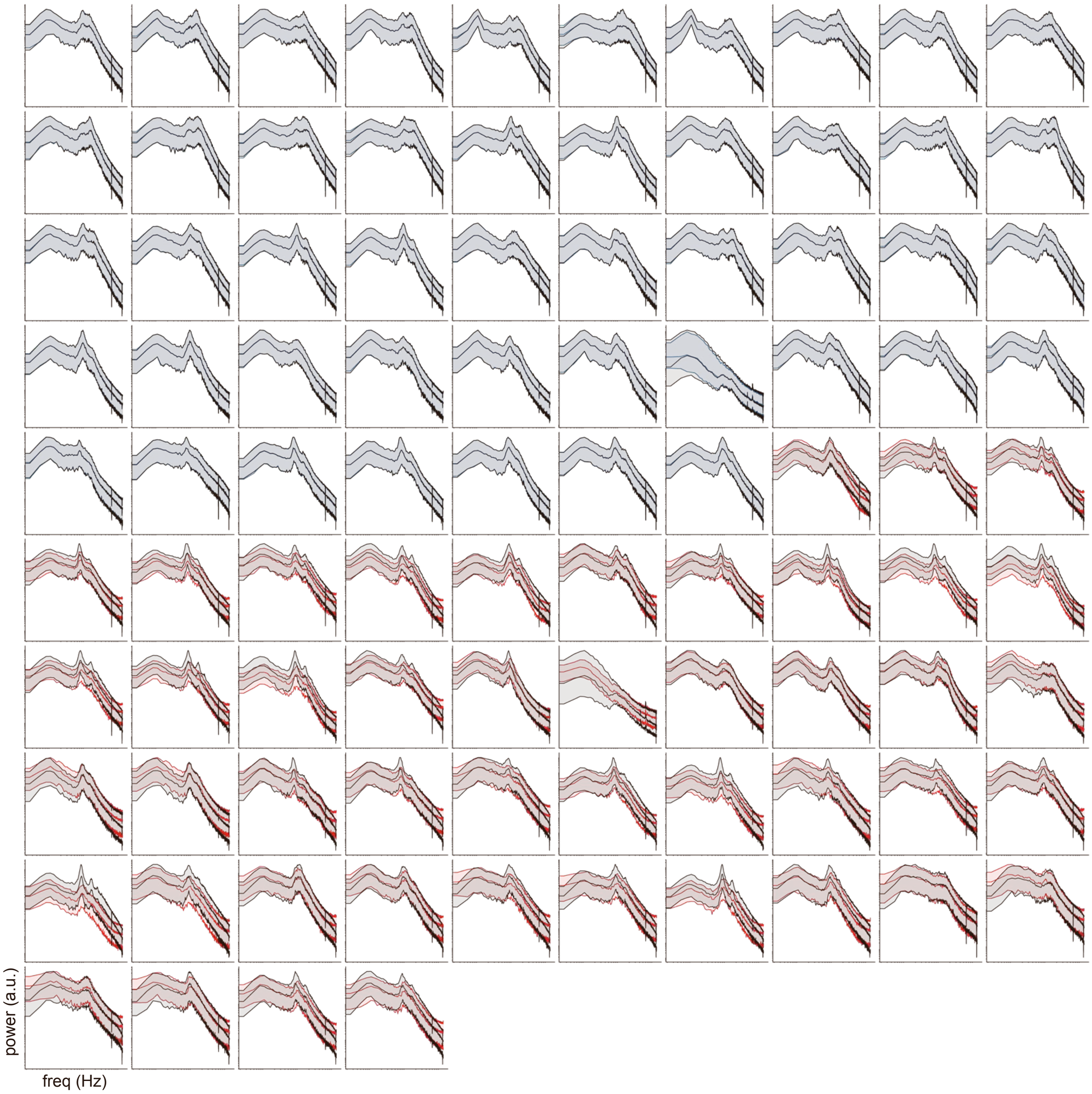

Figure S8: **Full spectra of P01 from the AJILE12 dataset.** The LFP traces consist of 94 channels. Here, we imputed the second half of the channels given the first half for all time series in the evaluation set. Median power as well as 10 %/90 % percentiles are shown.

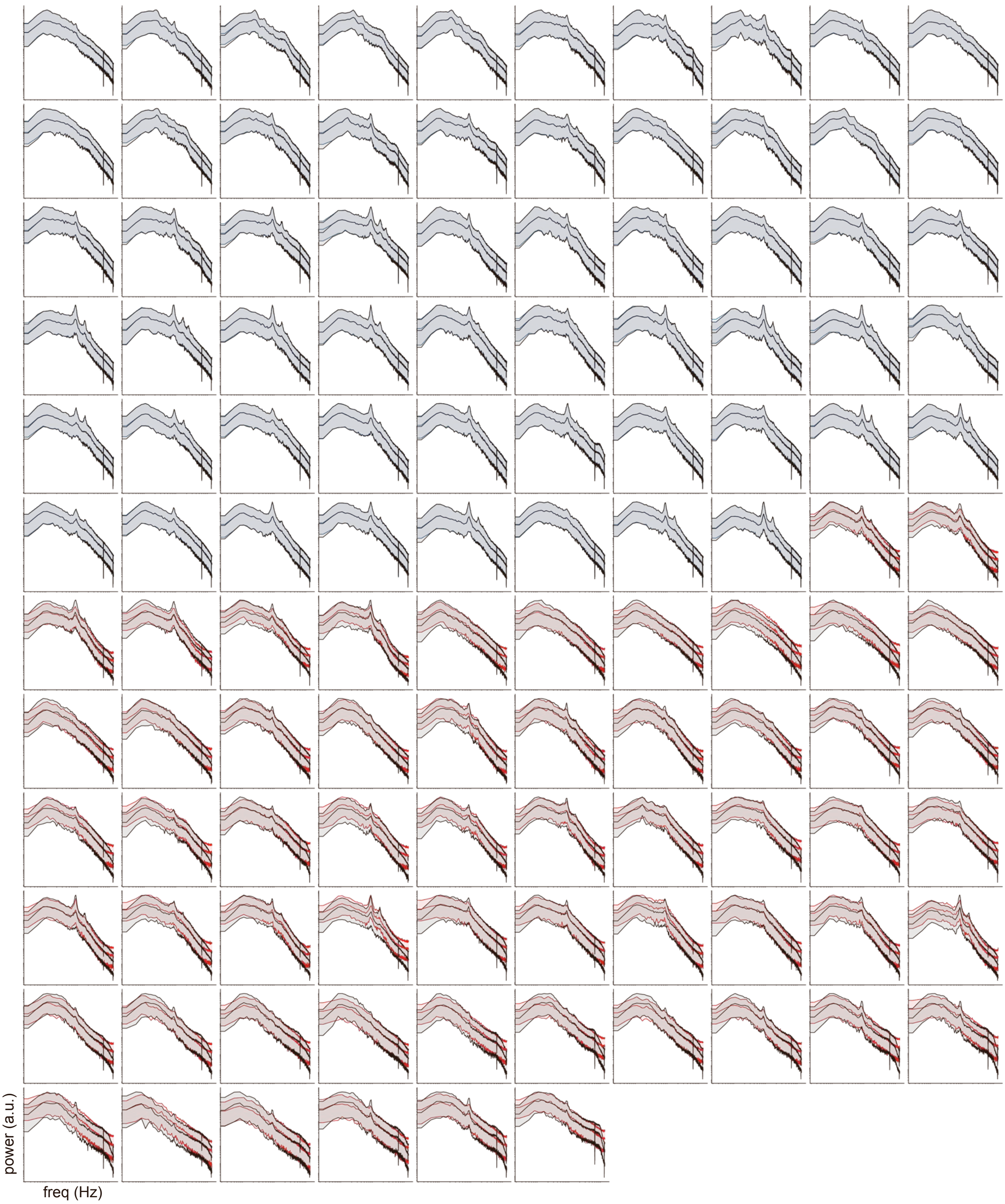

Figure S9: **Full spectra of P12 from the AJILE12 dataset.** The LFP traces consist of 126 channels. Here, we imputed the second half of the channels given the first half for all time series in the evaluation set. Median power as well as 10 %/90 % percentiles are shown.

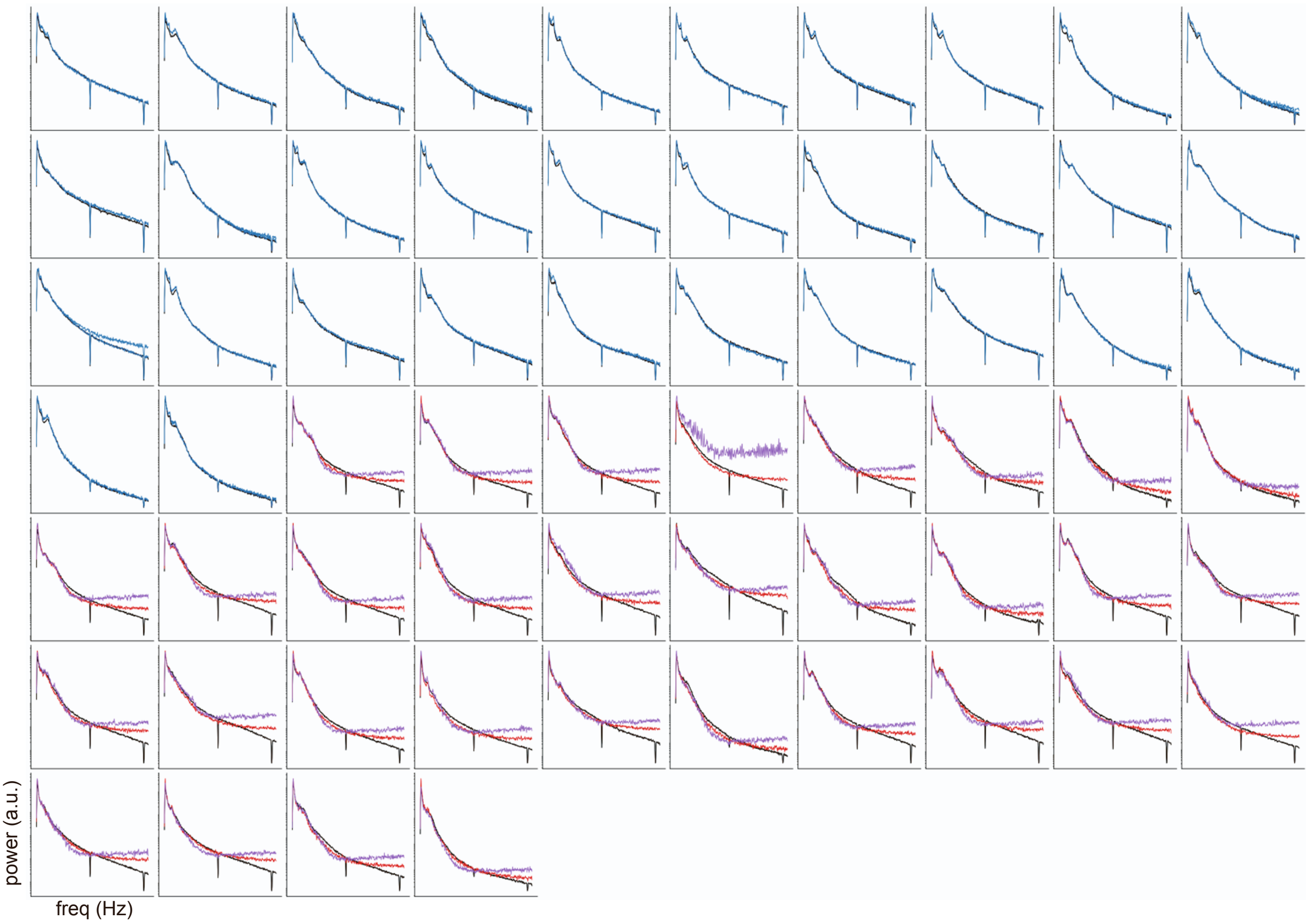

Figure S10: **OU versus white noise diffusion process for P01.** Full (semi-log) median spectra of P01 from the AJILE12 dataset. The second half of channels was imputed given the first half (blue) using a DDPM trained with either white noise (purple) or OU noise (red).

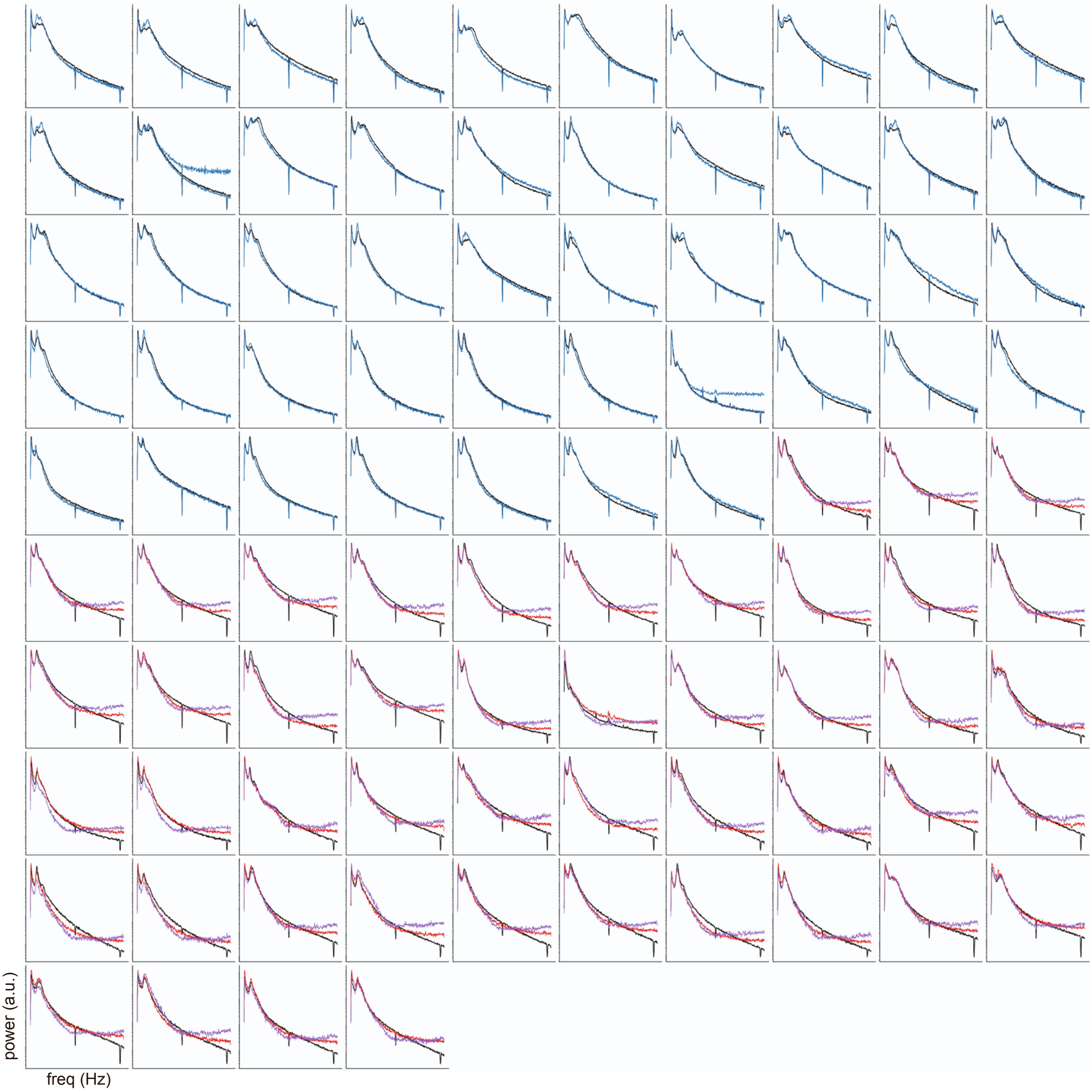

Figure S11: **OU versus white noise diffusion process for P07.** Full (semi-log) median spectra of P07 from the AJILE12 dataset. The second half of channels was imputed given the first half (blue) using a DDPM trained with either white noise (purple) or OU noise (red).

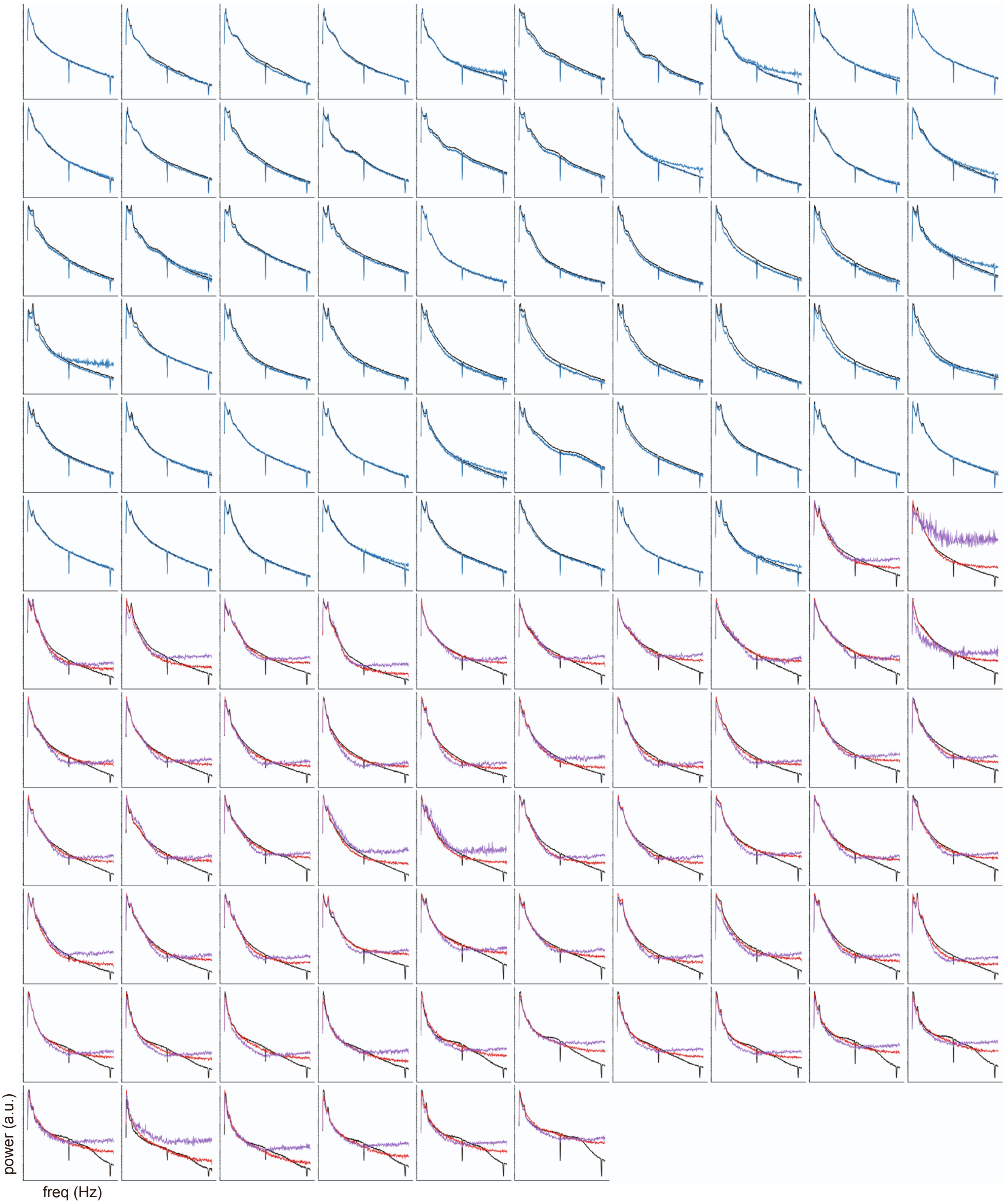

Figure S12: **OU versus white noise diffusion process for P12.** Full (semi-log) median spectra of P12 from the AJILE12 dataset. The second half of channels was imputed given the first half (blue) using a DDPM trained with either white noise (purple) or OU noise (red).

References

1. Biloš, M., Rasul, K., Schneider, A., Nevmyvaka, Y., and Günnemann, S. (2023). Modeling temporal data as continuous functions with process diffusion. International Conference on Machine Learning *202*, 2452–2470.

2. Talukder, S., Sun, J. J., Leonard, M., Brunton, B. W., and Yue, Y. (2022). Deep neural imputation: A framework for recovering incomplete brain recordings. NeurIPS 2022 Workshop on Learning from Time Series for Health.

3. Cazelles, E., Robert, A., and Tobar, F. (2020). The Wasserstein-Fourier distance for stationary time series. IEEE Transactions on Signal Processing *69*, 709–721.

4. Aznan, N. K. N., Atapour-Abarghouei, A., Bonner, S., Connolly, J. D., Al Moubayed, N., and Breckon, T. P. (2019). Simulating brain signals: Creating synthetic eeg data via neural-based generative models for improved ssvep classification. In: 2019 International joint conference on neural networks (IJCNN). (1–8).

5. Kingma, D. P., Welling, M. et al. (2019). An introduction to variational autoencoders. Foundations and Trends in Machine Learning *12*, 307–392.

6. Bredell, G., Flouris, K., Chaitanya, K., Erdil, E., and Konukoglu, E. (2023). Explicitly minimizing the blur error of variational autoencoders. International Conference on Learning Representations *abs/2304.05939*.

7. Kodali, N., Abernethy, J., Hays, J., and Kira, Z. (2017). On convergence and stability of GANs. Preprint at arXiv. https://doi.org/10.48550/arXiv.1705.07215.

8. Mescheder, L., Geiger, A., and Nowozin, S. (2018). Which training methods for GANs do actually converge? International conference on machine learning (3481–3490).
