# Supplementary material for: Generating realistic neurophysiological time series with denoising diffusion probabilistic models
Source: Patterns (N Y). 2024 Aug 29;5(9):101047. doi: 10.1016/j.patter.2024.101047 (PMC11573898; doi:10.1016/j.patter.2024.101047)
Supplement: Document S2. Article plus supplemental information [file mmc2.pdf]

Patterns

Article

Generating realistic neurophysiological time series with denoising diffusion probabilistic models

Graphical abstract

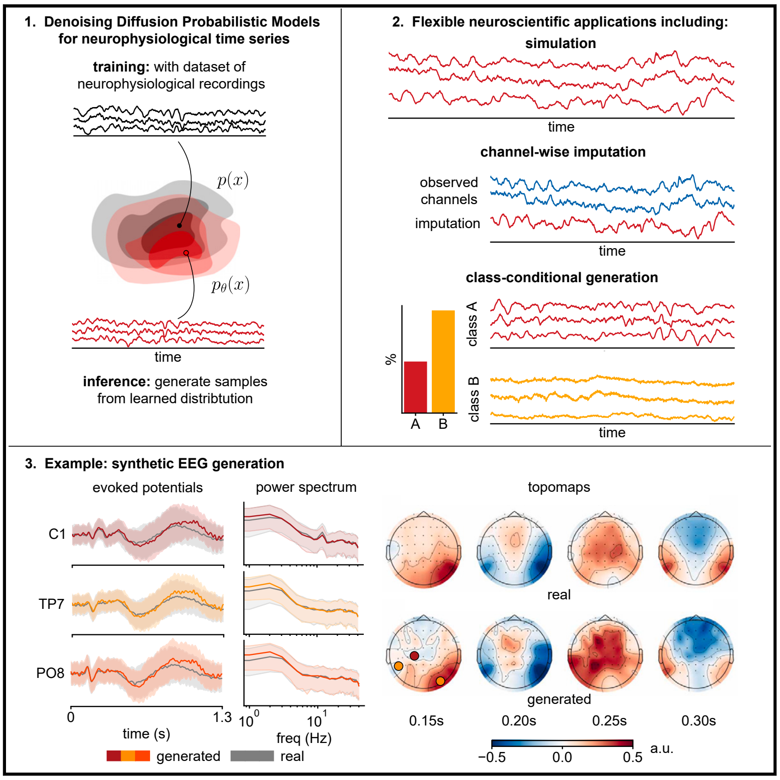

Highlights

- We present a denoising diffusion-based deep generative model for brain signals
- Our model accurately generates different data types, including EEG, ECoG, and LFP
- Data can also be generated while considering additional information, such as brain state
- Trained models and synthetic data facilitate several neuroscientific applications

Authors

Julius Vetter, Jakob H. Macke, Richard Gao

Correspondence

julius.vetter@uni-tuebingen.de (J.V.), jakob.macke@uni-tuebingen.de (J.H.M.), r.dg.gao@gmail.com (R.G.)

In brief

This study presents a denoising diffusion model tailored to neurophysiological time series. The model is able to generate a variety of neurophysiological time series, including electroencephalography (EEG), electrocorticography (ECoG), and local field potential (LFP) recordings, with high fidelity. The generated data accurately capture statistics of interest in neuroscience. In addition, the diffusion model can incorporate additional information into the generation process, facilitating several scientific and clinical applications, such as missing-data imputation or brain-state classification.

Vetter et al., 2024, Patterns *5*, 101047
September 13, 2024 © 2024 The Author(s). Published by Elsevier Inc.
https://doi.org/10.1016/j.patter.2024.101047

CellPress

Patterns

CellPress
OPEN ACCESS

Article

Generating realistic neurophysiological time series with denoising diffusion probabilistic models

Julius Vetter,[1,4,*] Jakob H. Macke,[1,2,3,*] and Richard Gao[1,3,*]
[1]Machine Learning in Science, University of Tübingen and Tübingen AI Center, Tübingen, Germany
[2]Max Planck Institute for Intelligent Systems, Tübingen, Germany
[3]These authors contributed equally
[4]Lead contact
*Correspondence: julius.vetter@uni-tuebingen.de (J.V.), jakob.macke@uni-tuebingen.de (J.H.M.), r.dg.gao@gmail.com (R.G.)
https://doi.org/10.1016/j.patter.2024.101047

THE BIGGER PICTURE Diffusion models are a class of machine learning models that can generate high-fidelity synthetic data. In recent years, they have had a profound impact on engineering and science, revolutionizing image and audio synthesis and advancing our ability to model scientific data. Capitalizing on this, we have designed denoising diffusion probabilistic models (DDPMs) for modeling brain recordings, such as electroencephalography (EEG), electrocorticography (ECoG), and local field potential (LFP) recordings. The ability to accurately generate realistic neurophysiological data has many applications in experimental, clinical, and computational neuroscience, including simulation, missing-data imputation, brain-state classification, and more, which we demonstrate in the present study.

SUMMARY

Denoising diffusion probabilistic models (DDPMs) have recently been shown to accurately generate complicated data such as images, audio, or time series. Experimental and clinical neuroscience also stand to benefit from this progress, as the accurate generation of neurophysiological time series can enable or improve many neuroscientific applications. Here, we present a flexible DDPM-based method for modeling multichannel, densely sampled neurophysiological recordings. DDPMs can generate realistic synthetic data for a variety of datasets from different species and recording techniques. The generated data capture important statistics, such as frequency spectra and phase-amplitude coupling, as well as fine-grained features such as sharp wave ripples. Furthermore, data can be generated based on additional information such as experimental conditions. We demonstrate the flexibility of DDPMs in several applications, including brain-state classification and missing-data imputation. In summary, DDPMs can serve as accurate generative models of neurophysiological recordings and have broad utility in the probabilistic generation of synthetic recordings for neuroscientific applications.

INTRODUCTION

Statistical models that can accurately reconstruct complex datasets are useful in many areas of science. These so-called generative models allow scientists to interpret and analyze statistical dependencies between data features, as well as between data and mechanistic latent variables. Such models further grant the ability to generate realistic synthetic data, which are valuable in their own right, especially when it is possible to incorporate (or "condition on") additional information or observations into the model. Examples of applications include imputing missing data given observed data, forecasting the future given past data, creating synthetic datasets as input to a simulator, and replacing or augmenting scarce training data for subsequent machine learning models—all of which rely on a (conditional) generative model that can produce realistic synthetic data.

The generation of synthetic neurophysiological recordings has received much attention over the years, predominantly with a focus on generating spike trains with a prespecified correlation structure.[1–3] More generally, time series surrogate methods[4,5] can be used to generate synthetic neurophysiological recordings that mimic the real data in a well-defined set of features but lose complex, nonlinear features by design. Other works have employed deep neural networks as nonlinear encoding models of

This is an open access article under the CC BY license (http://creativecommons.org/licenses/by/4.0/).

brain response, for example, predicting electroencephalography (EEG)[6] response from viewed images. However, they are deterministic by design and cannot be used to probabilistically simulate neural time series, nor can they be used without conditioning information such as visual stimuli.

Recently, probabilistic generative models that leverage deep neural networks have made a significant impact across various domains, transforming the way we approach tasks such as image and audio generation[7–9] and time series imputation[10] as well as scientific applications ranging from molecular design[11] to black hole imaging.[12] Neuroscience research, in particular, has benefited from various types of deep generative models. For example, variational autoencoders (VAEs) have been applied to infer low-dimensional representations of single-trial neural population dynamics,[13] while generative adversarial networks (GANs) have been used for the task of spike-train generation[14,15] and EEG generation,[16–18] as well as to decode images from single neuron and fMRI data.[19,20]

Denoising diffusion probabilistic models (DDPMs)[21]—or diffusion models for short—have recently become the generative model of choice thanks to their superior performance compared to VAEs and GANs in many applications.[8,22,23] They have also been applied in neuroscientific settings, such as leveraging latent diffusion models[9] to predict viewed images from fMRI data in a neural decoding context.[24,25] However, diffusion models have been underexplored for the realistic generation of a type of data that is ubiquitous in neuroscience: multivariate and densely sampled electrophysiological time series. Such recordings include noninvasive scalp EEG as well as intracranial electrocorticography (ECoG) and local field potential (LFP), which are routinely recorded during scientific research and clinical brain monitoring.

A general-purpose model that can conditionally synthesize such neurophysiological data would be valuable in many scenarios. For example, brain recordings often contain missing values due to sensor noise or misplacement,[26] which limits their use in neuroscience-specific applications and can lead to them being discarded altogether. Such practical problems are exacerbated by the fact that individual neurophysiological datasets are often limited in availability due to restrictions in clinical contexts. Deep generative models for time series can partially alleviate these problems by providing synthetic data, for example, to facilitate the training of a brain-computer interface (BCI) or as input data for scientific simulations with reduced privacy concerns, or by imputing missing channels in high-dimensional recordings.[27–30] While diffusion models have been used to model complex high-dimensional time series in a variety of settings,[23] including probabilistic imputation[31,32] and forecasting,[33,34] and more specific applications in neuroscience, such as data augmentation for seizure classification,[35] their general utility and performance in modeling neurophysiological recordings have not been explored.

In this work, we demonstrate that diffusion models can accurately generate multivariate and densely sampled continuous neurophysiological recordings and further showcase their utility in a variety of neuroscience-specific applications. In particular, we use deep neural networks with structured convolutions,[36] a class of highly expressive sequence-to-sequence models,[37,38] and Ornstein-Uhlenbeck (OU) processes as diffusion processes[34] to improve the modeling of such time series with DDPMs. Our trained models are capable of producing synthetic recordings with realistic power spectra and even complex features such as sharp wave ripples (SWRs) and cross-frequency coupling. By conditioning on observed values and other behavioral or experimental variables, synthetic recordings automatically capture cross-area interactions between many channels, which are used to impute missing channels and improve performance in a human BCI application at test time. Finally, DDPMs provide additional benefits accessible to generative models, enabling behavioral state classification and outlier detection without additional network training. The code for our method and to reproduce our existing results is available at https://github.com/mackelab/neural_timeseries_diffusion.

RESULTS

DDPMs for neurophysiological time series

We use DDPMs[21] to learn generative models of electrophysiological recordings. DDPMs are defined by a forward (noising) process and a reverse (denoising) process. The noising process successively adds random noise from a prespecified distribution (usually independent Gaussian) to the real data through a series of diffusion steps. The reverse process uses a deep neural network as a "denoiser," which is trained to reverse the forward process at each diffusion step (Figure 1A). To generate synthetic data, samples of random Gaussian noise are drawn and gradually denoised by the trained network (Figure 1A, box, top to bottom) over the same number of diffusion steps, resulting in synthetic data that follow the statistical distribution of the real data (i.e., unconditional generation or simulation; Figure 1B). The training and inference procedure can also incorporate additional information through conditioning. For example, when real data from other channels are given alongside the initial noise sample for the generated channel, the denoising process is guided to generate synthetic data that are likely given the other channels, which can be used to perform imputation of missing channels or interpolation (Figure 1C). Similarly, when behavioral or experimental state variables are included during training, they can be used to guide the denoising process during time series generation (Figure 1D). In addition, such a conditionally trained model can also act as a classifier or outlier detector since it can be queried for the likelihood of observing a data sample given either condition.

We follow the standard DDPM training and inference procedure[21] while incorporating two recent innovations in time series modeling: first, since neurophysiological time series are densely sampled (e.g., sampling rate of 500 Hz or more), learning long-range dependencies over hundreds of time points in just 1 s of data is a nontrivial challenge. We address this problem by using structured convolutions in the denoising network, which use a collection of multiscale convolution kernels that have shown performance and efficiency improvements on long-range time series tasks.[36] Second, neurophysiological recordings of all modalities (EEG, ECoG, LFP) follow a $1/f$-like power law in the frequency domain under a range of behavioral and brain states,[39–42] in contrast to the flat spectrum of the Gaussian white noise commonly used in DDPMs. Therefore, we sometimes use the OU process (i.e., colored noise) in the forward noising

CellPress
OPEN ACCESS

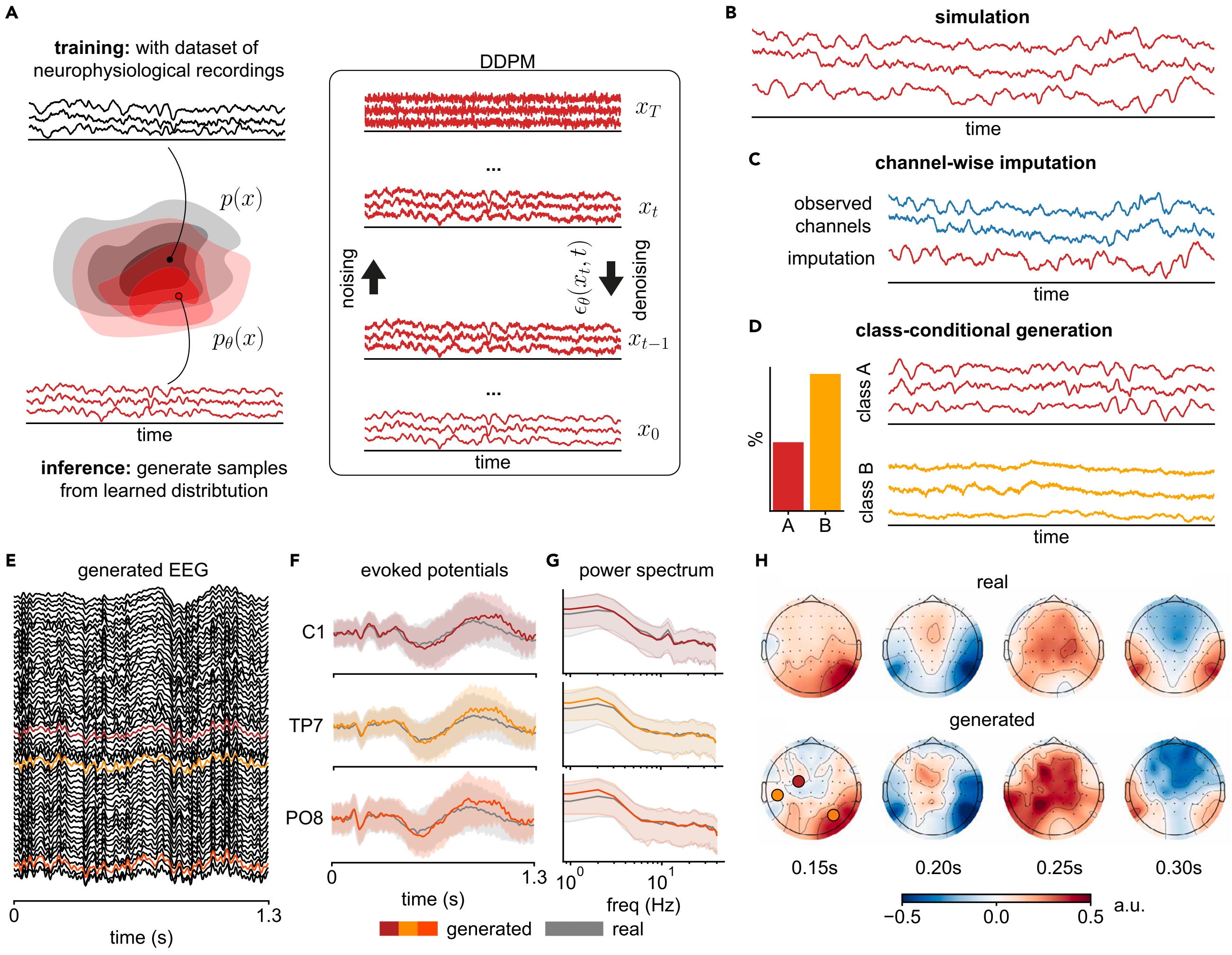

Figure 1. Overview of diffusion models for neurophysiological recordings and subsequent applications and an example of DDPM-generated EEG signal

(A) A denoising diffusion probabilistic model (DDPM) $p_\theta(x)$ is trained on a dataset of neurophysiological recordings. It attempts to generate samples from the data distribution $p(x)$, underlying the training data, by successively denoising samples from a prespecified Gaussian distribution, using a neural network $\epsilon_\theta(x_t, t)$ as the denoiser. In the context of neurophysiological recordings, DDPMs can be used for various different tasks.

(B–D) Examples include (B) simulation of neurophysiological recordings, (C) imputation of missing values in these recordings, and (D) class-conditional generation of recordings from different experimental conditions or brain states. Since DDPMs allow the computation of likelihoods, the class-conditional model can also be used to perform tasks like classification or outlier detection.

(E) An example DDPM-generated trial of 56-channel EEG.

(F–H) Trial average of three channels show close overlap between real (gray) and generated (colored) (F) evoked potentials (mean and standard deviation across trials), (G) power spectra (median and 10%/90% percentiles), and (H) spatiotemporal relationships reflected in scalp topography.

process[34] to incorporate this prior knowledge and empirically demonstrate improvements over the standard white noise. Full details on DDPMs, as well as our denoising network architecture and parameterization of the OU process, can be found in the experimental procedures.

As a first demonstration, we train a diffusion model on a single participant's EEG recorded during a trial-structured task (BCI Challenge @ NER 2015, Margaux et al.[43]). The trained DDPM is able to simulate (i.e., unconditionally generate) realistic single-trial EEG data (Figure 1E) and capture features in the real trial-averaged time series and power spectra (Figures 1F and 1G), in particular the evolution of the evoked potentials and a 12 Hz oscillation in the C1 channel. In addition, spatiotemporal relationships across the entire scalp are preserved, as shown in the topographic plots over time (Figure 1H). This introductory demonstration illustrates how DDPMs can realistically generate high-dimensional neurophysiological time series, and in the following sections, we further evaluate and exploit this capability on a variety of datasets and applications.

Overview of DDPM applications: Experiments and datasets

Beyond the first example, we apply our model to three different datasets to test whether diffusion models can generate realistic

synthetic brain recordings in different settings and demonstrate their utility in a variety of tasks relevant to neuroscience. On all three datasets, DDPMs accurately capture the statistics of real recordings—in particular, the distribution of channel-wise power spectral densities (PSDs), as well as the multivariate cross-spectrum—across species, recording modalities, and brain areas. Critically, the trained models do not simply overfit to or memorize samples from the training set, which we confirm by measuring the pairwise distance between generated and training data in the time and frequency domains (Figure S4). We show that DDPMs are a general-purpose generative model that accurately capture idiosyncratic features in each of the three datasets and can be applied in a variety of tasks with minor modifications to network architecture or training procedure.

The first dataset consists of 3-channel LFP recordings from rat prefrontal cortex, thalamus, and hippocampus.[44] These recordings contain SWRs that exhibit complex cross-frequency and cross-channel dependencies, which are correctly captured by our model in both simulation and imputation settings. The second dataset consists of human ECoG recorded during naturalistic behavior from 12 participants undergoing epilepsy monitoring (AJILE12, Peterson et al.[45]). In addition to accurately generating high-channel-count ECoG data, we show that DDPMs can sensibly impute corrupted or missing channels in an artificially induced missing-data setting (similar to Talukder et al.[30]), which substantially improves performance in a neural decoding task compared to a baseline (mean) imputation. The third dataset consists of whole-brain ECoG recordings from a macaque monkey under two different brain states (awake and anesthetized). DDPMs can conditionally generate recordings given one of these two states, as well as evaluate class-specific likelihoods, which in turn can be used to perform classification and outlier detection. The rest of this section presents detailed results from each of the three experiments.

DDPM captures cross-frequency and cross-region dependencies of SWRs

In the first experiment, we apply our DDPM to model LFPs recorded from freely behaving rats (sampled at 600 Hz) in three locations: medial prefrontal cortex (mPFC), hippocampal CA1 region, and the thalamic nucleus reuniens (RE).[46] These recordings contain neurophysiological features of interest, such as slow oscillations, spindles, and SWRs, which are region specific and occur with a specific phase relationship relative to one another.[44] Thus, we aim to generate synthetic data and assess whether our model is able to correctly capture the single-channel voltage distribution and power spectrum as well as complex features such as phase-amplitude coupling (PAC) between slow oscillations and ripples across channels. In addition, to compare the fidelity of generated recordings using different types of generative models, we also train a VAE[47] and a GAN[48] from a recently published study on generative modeling of electrophysiological brain recordings.[16] Furthermore, we improve both models by incorporating more competitive encoder/decoder and generator/discriminator architectures based on the structured convolutions used in our denoiser architecture.

After training models on 3,480 2-s time series, we generate the same number of synthetic samples for comparison. Visually, DDPM-generated traces look indistinguishable from real data and contain clearly visible SWRs (example in Figure 2B). Across all generated samples, the distribution of voltage values and PSDs between real and synthetic data match closely in all three regions (Figure 2C), and components such as 60 Hz line noise are also reproduced correctly. For the VAE and GAN baselines, only the models that use our architectures based on structured convolutions are capable of producing visually consistent samples (see Figure S1). Nevertheless, VAE samples are overly smooth (i.e., low-frequency dominated), while GAN samples contain frequency characteristics not found in the real data and fail to capture finer details (Figures S2 and S3).

To assess whether our DDPM can reproduce more complex cross-regional and ripple-specific features in the real data, we measure the degree of PAC between the amplitude of the CA1 ripple band (100–275 Hz) and the phase of delta (1–4 Hz), theta (4–9 Hz), and spindle (6–14 Hz) frequencies in the mPFC. We additionally isolate this analysis to the actual occurrences of ripples by thresholding the ripple band power and computing a "phase count coupling" histogram between detected CA1 ripples and the instantaneous phase of the aforementioned driver frequencies in the mPFC (similar to Varela and Wilson[44]). Overall, we see that ripples in both generated and real data have remarkably similar phase preferences and, for all three frequencies in the mPFC, peak between -45° and 45° (Figure 2D). As a baseline comparison, we also created channel-wise surrogates[5] that preserve the single-channel PSD but do not show any preferred phase coupling. Similarly, the recordings generated by the VAE and GAN do not exhibit the correct PAC (Figures S2 and S3).

While the above demonstrates that DDPM-generated samples reproduce the correct temporal relationships between CA1 ripples and slower fluctuations in the mPFC, we further investigate whether SWRs are correctly captured by our model by conditionally generating (i.e., imputing) CA1 traces given real mPFC and RE traces for a set of held-out evaluation time series. In other words, when provided with the other channels as context, can our model correctly infer when a ripple would have occurred in CA1? We use a technique known as "inpainting" to impute the CA1 channel.[49] This technique allows DDPMs to fill in missing values by guiding the diffusion process along observed values in the other dimensions (i.e., channels). To evaluate performance for a given (held-out) evaluation sample, we check if both the real and imputed CA1 traces contain a ripple by thresholding the ripple band power and then compute the F1 score for this classification task across the whole evaluation set. To test whether our model is better than chance at generating CA1 ripples given the appropriate context, we perform a permutation test by shuffling the binary labels of the real CA1 data (i.e., "was there a ripple or not?") and recomputing the F1 score.[50]

We provide an example of a successfully imputed ripple (Figure 2E), where the generated CA1 trace contains a ripple at exactly the same time as when the real ripple occurred around the 800 ms mark. Repeating the imputation procedure 100 times on the same mPFC and RE traces, we see that the average ripple band power in the imputed CA1 traces is highest at the time that coincides with when the real ripple occurred (Figure 2F). Across all evaluation samples, our model conditionally generates ripples

CellPress
OPEN ACCESS

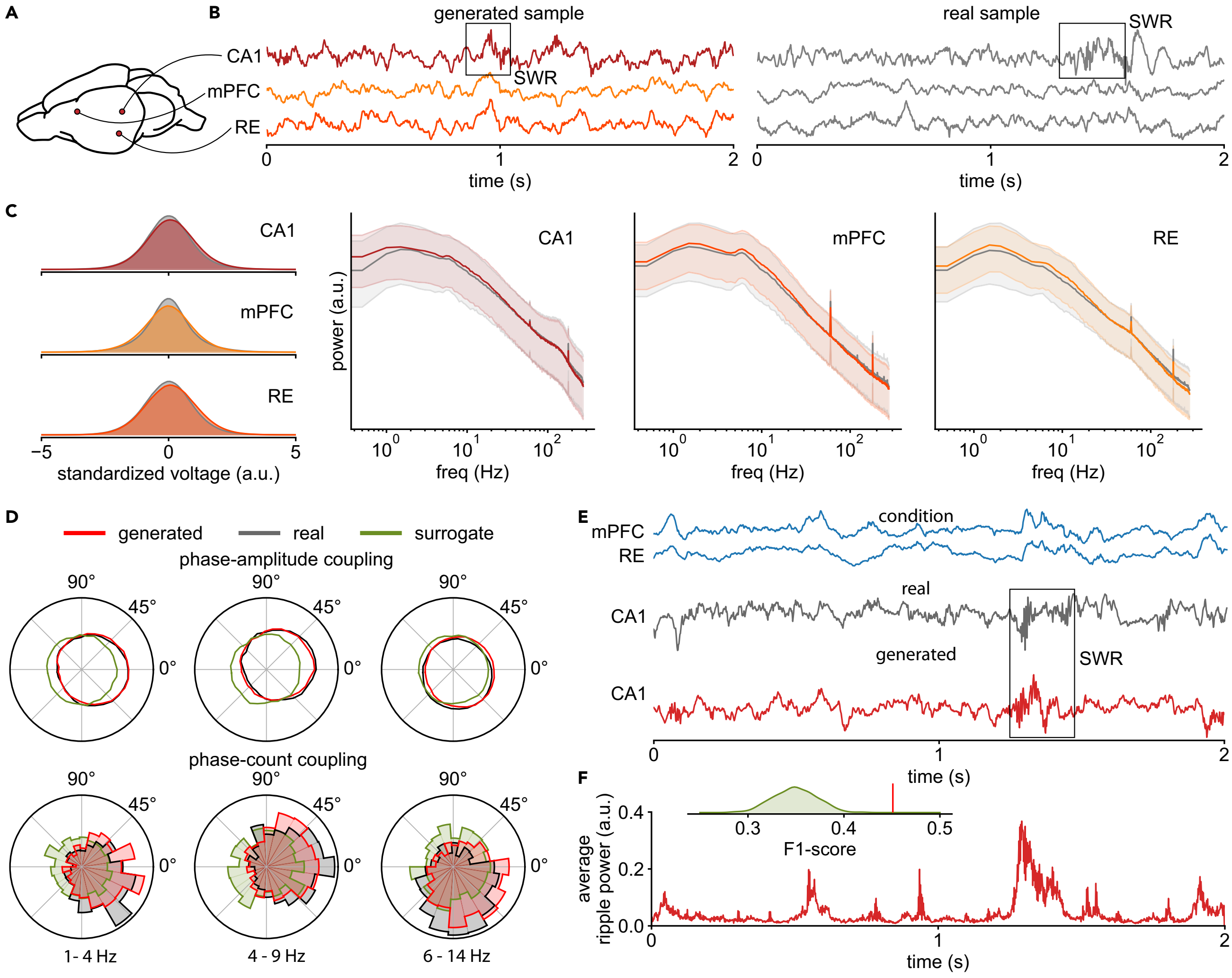

Figure 2. DDPM captures rat cortical-hippocampal LFP dynamics

(A) Schematic of rat brain showing recording locations: CA1, mPFC, and RE.

(B) Example of time series generated by our model (left) and a real sample (right). Both examples contain SWRs.

(C) Marginal distributions over the standardized voltage of each channel for both real and generated data (left), as well as median and 10%/90% percentiles of real and generated power spectra for each channel (right).

(D) Phase-amplitude and phase-count coupling of CA1 ripples for different driver frequencies in the mPFC for real, surrogate, and DDPM-generated data.

(E) Example of conditionally generated CA1 trace given real mPFC and RE traces. The real and conditionally generated traces contain a ripple in the same position.

(F) Average power in the SWR frequency band (100–275 Hz) over 100 samples from the model (conditioned on the same mPFC and RE traces as in E) has a large peak at the same position, indicating that many SWRs are generated at this location. Inset: across the evaluation dataset, the prediction performance (“is an SWR present in both the real and predicted time series?”) measured in terms of F1 score is significantly different from the distribution of scores for randomly permuted predictions.

significantly better than chance (F1 = 0.45, $p_{perm} < 0.001$; Figure 2F, inset). Thus, our model successfully captures within-channel temporal characteristics, as well as cross-frequency and cross-channel relationships observed in rat LFPs during SWR occurrences, and can therefore be used for probabilistic generation or simulation of highly complex brain recordings. This ability to produce high-quality simulations also extends to more high-dimensional examples such as the 56-channel EEG data (Figures 1E–1H) or the 128-channel macaque ECoG data (Figure S6).

DDPM-generated imputations improve neural decoding with missing data

In our second experiment, we use the AJILE12 dataset,[45] which consists of ECoG recordings from twelve human participants during naturalistic behavior while undergoing epilepsy monitoring (electrode coverage of one example participant is shown in Figure 3A). Using an artificially induced missing-data scenario (following a similar setup as in Talukder et al.[30]), where only a subset of the recorded channels are observed and the goal is to predict the missing channels from the observed ones, we

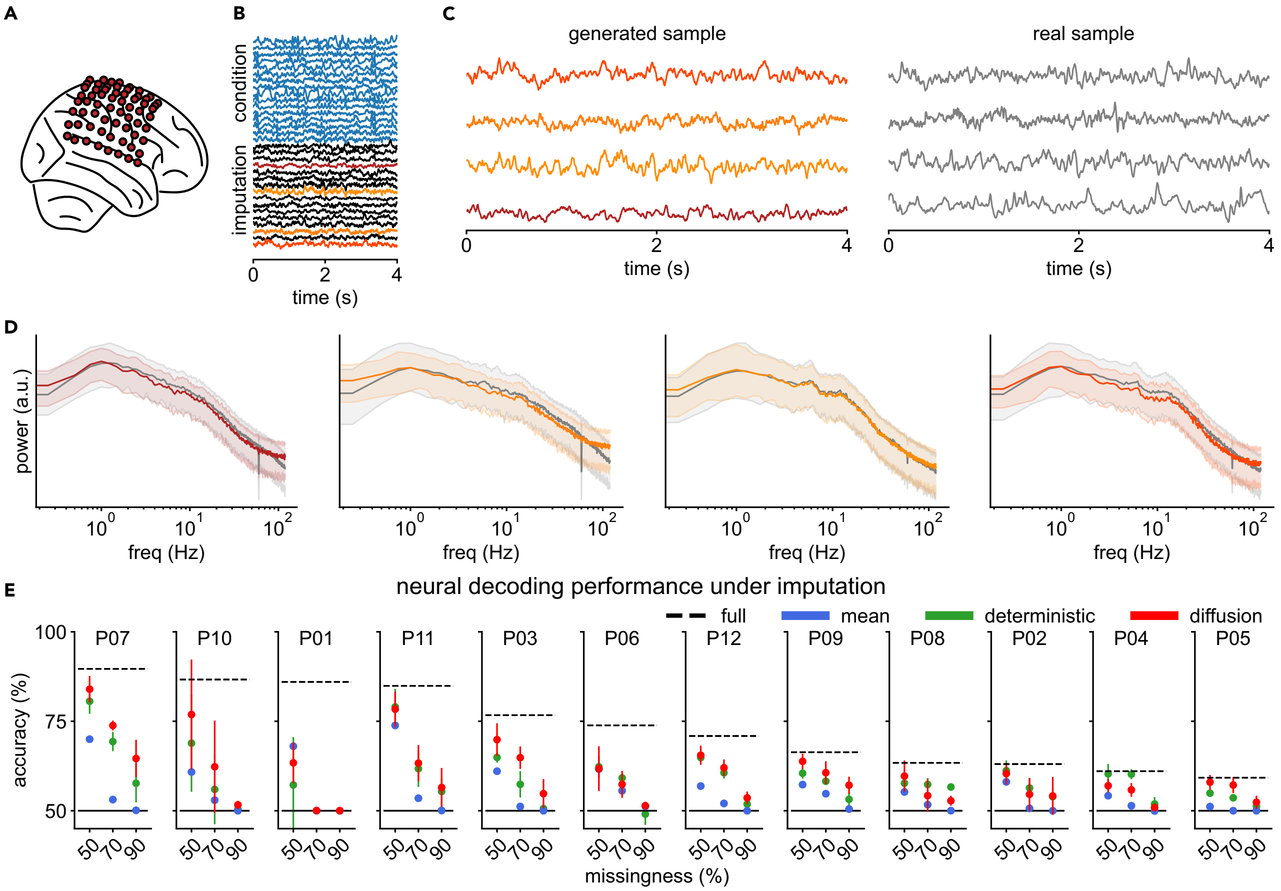

Figure 3. DDPM-generated imputations of human ECoG recordings improve BCI decoding

(A) Electrode layout of participant P07 from the AJILE12 dataset.

(B) Example imputation of a 4-s window from P07. The first 32 channels are used as conditioning information (blue), while the remaining 32 channels are imputed (conditionally generated); four are highlighted and shown in (C).

(C) Four randomly selected channels from the imputation (left) together with the corresponding real sample (right).

(D) Median and 10%/90% percentiles of real and generated power spectra for the four channels.

(E) Test performance of the neural decoding model for each of the 12 participants with fully observed data (dashed) as well mean-imputation (blue), deterministic-neural-network-based imputation (green), and DDPM-based imputation (red) under different amounts of missingness. Participants are sorted by the decoder performance on fully observed data. Mean and standard deviation over five different randomly drawn missing channel configurations are shown.

demonstrate how diffusion-based imputation can subsequently improve classifier performance in a neural decoding task.

For each participant, we train a diffusion model on all channels from their first 6 days of recording, with the number of 4-s-long training time windows varying between 148 and 1,512 per participant. Unlike the model used for rat LFP data, the diffusion model for each AJILE participant is trained to directly capture the conditional distribution of missing channels given observed channels. This is achieved by randomly dropping out channels during training and conditioning on the remaining channels to perform imputation. Conditional training can further improve imputation performance over the previously used unconditional training.[31] Details on the dataset, network architecture, and conditional versus unconditional training are given in the experimental procedures.

After training the model, we test its ability to impute "missing" channels of held-out evaluation samples from the seventh day of recording by generating synthetic data for half the channels while conditioning on real data from the other half (i.e., 50% missingness; Figure 3B). Conditionally generated synthetic traces look visually similar to real data in the corresponding channels (Figure 3C, 4 out of 64 channels highlighted), while synthetic PSDs closely match those of the real data for the imputed channels across all evaluation time series (Figures 3D, S8, and S9), demonstrating that DDPMs can be used for participant-specific imputation even with a substantial amount of channels missing. For the AJILE12 dataset, the quality of imputed traces is improved by using OU processes instead of white noise (experiments on the effect of using the OU process versus white noise are described in the supplemental information; Figures S10–S12).

To further quantify the quality of the imputations, we compute the correlations between the ground truth and imputed channels (as in Talukder et al.[30]). For each participant, 50%, 70%, and 90% of the channels are randomly dropped from all time series of the evaluation set and then probabilistically imputed using

the trained diffusion model (as in Figure 3B). This procedure is repeated with five different random seeds for each dropout rate. We also compare the diffusion-based imputation to a mean-imputation baseline—a strategy often employed in such neural decoding paradigms when faced with missing data.[30] Furthermore, to provide a stronger baseline, we perform neural-network-based imputation, where we use a regression network based on our denoiser architecture with structured convolutions and train it to perform deterministic imputation by minimizing the mean-squared error (an example of deterministic imputation is shown in Figure S5).

For a dropout rate of 50%, the average channel-wise correlation across all participants and test signals is 0.27 for DDPM-generated samples. The imputation performance varies widely between participants, with the best one achieving an average correlation of 0.42 and the worst a correlation of 0.11. As expected, with increasing dropout rates (70%, 90%), the correlation further drops. In comparison, mean imputations have a correlation of zero by definition. With an average correlation of 0.50 for a dropout rate of 50%, the deterministic, neural-network-based imputations are more correlated to the true data than the probabilistic DDDP-based imputations (see numerical comparison in Table S1). However, as shown next, this does not necessarily translate into higher performance in neural decoding tasks.

To demonstrate the utility of diffusion-based imputation, we use a binary neural decoding task of classifying the 4-s-long time series that were recorded under rest from those recorded during movement. We use per-participant random forest classifiers that were also trained on the first 6 days of each participant's recording as decoders (similar to Peterson et al.[51]) and evaluate classification performance on fully observed evaluation time series to establish a performance upper bound (Figure 3E, dashed). We then classify the imputed time series using the random forest classifier and report the average and standard deviation across dropout random seeds (Figure 3E, red).

Across 12 participants and 3 missingness levels, we observe that diffusion-based imputation (Figure 3E, red) leads to better classification performance than mean imputation (Figure 3E, blue) in all participants, apart from P01 and P06. In addition, the DDPM-based, probabilistic imputations achieve a decoding accuracy that is competitive with the neural-network-based, deterministic imputations (Figure 3E, green): the increase in decoding accuracy over the mean imputations is larger for the DDPM-based imputations 8 out of 12 times for both 50% and 70% dropout rates and 9 out of 12 times for a 90% dropout rate (see a numerical comparison for all participants and dropout levels in Table S1).

Similarly, the DDPM compares favorably to the decoding performance of the deterministic imputations from the autoencoder-based models from Talukder et al.,[30] which are specifically tailored to perform imputation (numerical comparison in Table S1). Overall, like the channel-wise correlations between ground truth and imputed time series, the decoding performances after imputation appear highly participant dependent. Nevertheless, diffusion-based imputation significantly improves neural decoding accuracy, even with a significant proportion of channels missing.

Class-conditional DDPMs can generate and evaluate brain-state-dependent recordings

In our third experiment, we use the DDPM to model whole-brain surface ECoG from a nonhuman primate (macaque monkey) sampled at 1,000 Hz.[52] In this experiment, we model 12 randomly selected channels (out of 128; Figure 4A) during two distinct behavioral states: awake, a control condition where the animal is head and arm restrained but otherwise freely behaving, and anesthetized, where the animal is fully unconscious following the induction of general anesthesia. Our class-conditional diffusion model can accurately generate synthetic recordings under both states, which can be used to train a classifier without giving it access to real data. In addition, we can evaluate likelihoods under the learned generative model, which can be used for state classification and outlier detection of real recordings.

After training our model on a total of 870 1-s-long windows from both the awake and anesthetized conditions, we conditionally generate the same number of synthetic time series for evaluation. For both the awake and anesthetized conditions, the real and generated example time series are visually indistinguishable from each other (Figure 4B, 4 out of 12 channels are highlighted). Notably, synthetic data during the awake state contain more higher-frequency fluctuations and oscillations lasting several cycles, while abrupt lower-frequency fluctuations dominate during the anesthetized state. Across all 12 modeled channels, the PSDs of real and synthetic data closely match in both conditions (Figure 4C).

In addition to generating synthetic recordings in a state-dependent manner, class-conditional DDPMs can also evaluate the likelihood of recordings under each condition; that is, how likely is it to observe a given recording under awake versus anesthetized states? Using the difference of log likelihoods from our trained models to classify previously unseen evaluation time windows from either state, we achieve an evaluation accuracy of 83% on the balanced classification task. Additionally, when we evaluate likelihoods over a 1.5-h segment of the recording in a sliding-window manner, we observe that the difference in log likelihood between anesthetized and awake conditions is negative to start and then sharply increases after the induction of general anesthesia—even though the diffusion model does not have access to this information, nor was it trained on nearby segments of the recording (Figure 4D, training segments in blue). As time elapses, the difference of log likelihoods slowly decreases toward the "awake level" (negative) as the anesthesia wears off, demonstrating how such generative models can be applied for continuous state prediction.

Additionally, unlike with purely discriminative classifiers, the ability to evaluate likelihoods allows us to use our generative model to perform outlier detection, which we demonstrate through two experiments. In the first experiment, for all time series in the evaluation set, we replace half of the channels with white noise to simulate complete signal loss in some of the recording electrodes. In the second experiment, we instead reverse half of the channels time-wise, which is unlikely to happen in the real world but creates time series that are visually and statistically similar to the real data, thus representing a difficult out-of-distribution test scenario. Using the likelihood of the real and outlier data provided by the diffusion model as a score for classification, the area under the receiver operator

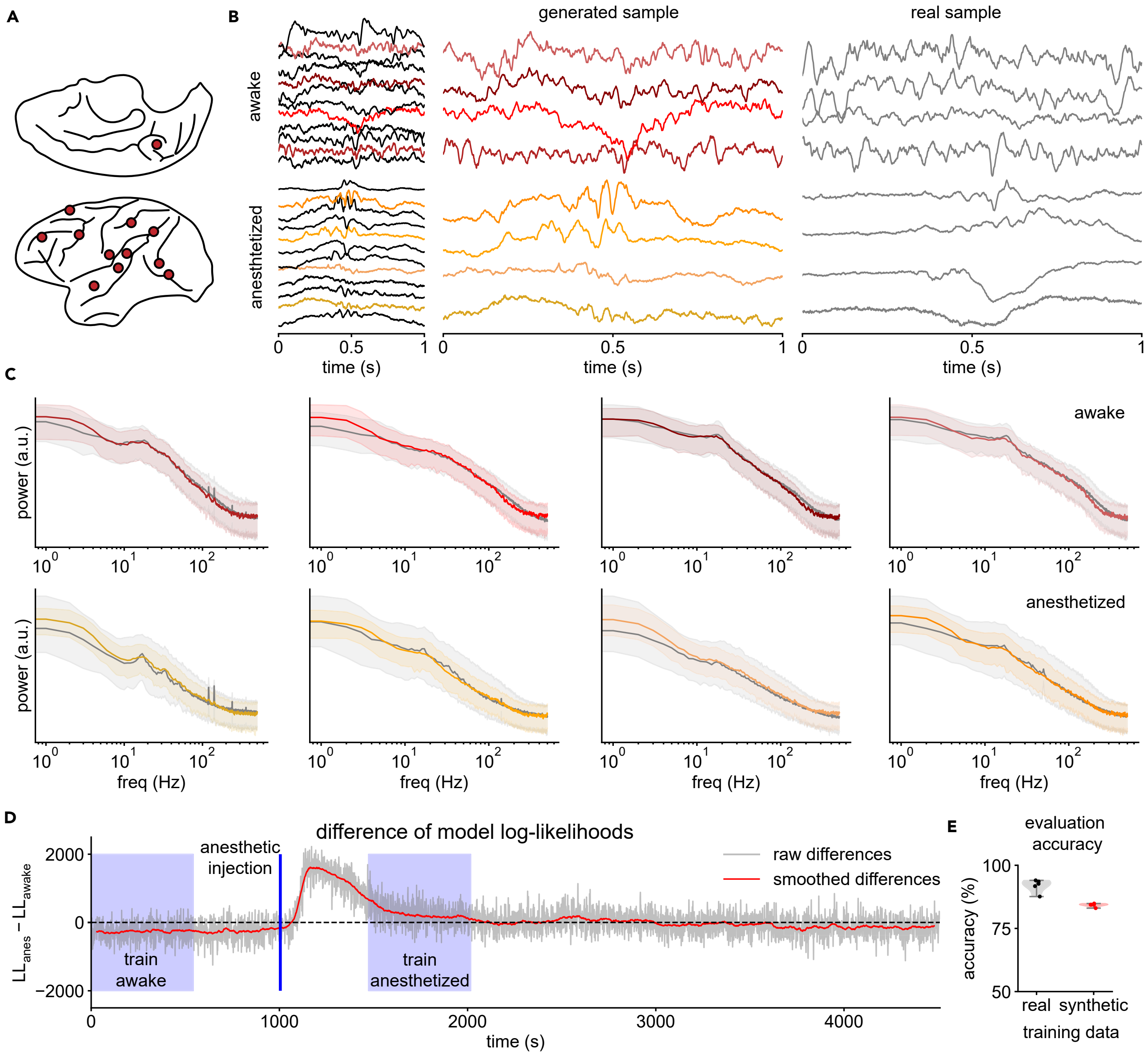

Figure 4. Conditional DDPM learns brain state-dependent macaque ECoG recordings
(A) Electrode locations in the macaque brain of the 12 randomly selected channels used for modeling.
(B) Examples of conditionally generated time series for both the awake and anesthetized conditions. Four randomly selected channels are shown in more detail (left) together with real samples (right).
(C) Median and 10%/90% percentiles of real and generated PSDs for the four channels stratified by condition.
(D) Difference of log likelihoods over 1.5 h of recording. A positive difference indicates a classification toward "anesthetized" and a negative difference toward "awake." Periods used for model training are shaded in blue.
(E) Real data test accuracy of a brain-state classifier trained on real (black) versus DDPM-generated synthetic data (red).

characteristic (AUC) for time-wise-flipped outliers is 0.63, while the AUC for white noise outliers is 1.0. When comparing pairs of time-wise flipped outliers with their corresponding real time series, the likelihood of the real time series is larger in 98% of the cases, demonstrating the model's ability to distinguish data that have the same marginal distribution per channel but are nevertheless out of distribution.

Finally, to further demonstrate the utility of using diffusion models to generate realistic neurophysiological recordings, we perform brain-state classification based on synthetic data. In many situations, the original data are sensitive due to privacy concerns, for example, when collected as a part of medical examination, and therefore cannot be publicly shared but may otherwise be useful for applications such as training of biometric algorithms. In such situations, synthetic recordings that preserve specific aspects of the recording while anonymizing others may be valuable. Here, we use the trained DDPM to generate synthetic data from awake and anesthetized conditions and train a

neural-network-based discriminative classifier (as described in Wang et al.[53]) solely on these synthetic data. The trained classifier is then evaluated on a held-out evaluation set of real recordings while never having seen real data before. Since the diffusion model captures the conditional distribution of the two classes well, the classifier trained on synthetic data achieves an accuracy of 84% when classifying real data (Figure 4E), which is close to the accuracy of a classifier trained on real data (92%). We thus demonstrate the ability to create artificial datasets that can be shared to facilitate further research with reduced privacy concerns.

DISCUSSION

Summary

Our experiments show that DDPMs, together with a flexible convolutional denoising network and the use of the OU processes, are a powerful tool that can accurately model highly multivariate and densely sampled neurophysiological data, providing features and, more importantly, realistic synthetic data useful for applications relevant to neuroscience. The power of DDPMs lies in their generality, as they provide a general-purpose generative model that can be used for many different applications ranging from unconditional (i.e., simulation) and conditional generation to imputation and likelihood evaluation.

We demonstrate these applications here on three different datasets of neurophysiological recordings with channel counts ranging from 3 to 128 channels from a variety of brain regions, recording modalities, and species. Overall, our models accurately capture data distributions in the time and frequency domains, as well as detailed cross-channel and cross-frequency dependencies like SWRs. On an imputation task, they achieve a performance that is on par with or better than tailored imputation methods for neurophysiological recordings. Finally, we show successful applications of class-conditional models in classification, outlier detection, and synthetic training data generation.

These features make DDPMs a viable tool for neuroscientists and clinicians studying the complex dynamics embedded in neurophysiological recordings. After successful training, DDPMs can be used to generate unlimited amounts of data as input to neuroscience simulators. When neurophysiological data are subject to strict privacy requirements, as may be the case with human clinical data, neuroscientists and clinicians can use DDPMs to generate artificial datasets that can be shared with collaborators with less concern. In addition, the accurate imputations provided by DDPMs can eliminate the need to discard large amounts of data or modify an already trained neural decoder. Finally, DDPMs output log likelihoods that provide users with a model to perform simple classification tasks or outlier detection at no additional training cost.

Other potential applications

Trained DDPMs can be used for a variety of other applications that we do not show in this work, such as training data augmentation, as well as imputation of more complex missingness patterns, which includes the task of time series forecasting.

Instead of completely replacing training datasets with artificial ones, as described in the data privacy use case (Figure 4E), it is possible to augment existing real training data with synthetic data generated by a DDPM to improve the performance of another model (e.g., a classifier). This additional training data could lead to performance improvements, especially if the relevant model is difficult to regularize and prone to overfitting. DDPMs can provide tailored noise-augmented data samples that capture complex relationships other surrogate methods may not. Throughout our experiments, DDPMs were robust to overfitting and able to achieve good sample quality with only a few hundred training time windows of 1- to 4-s long. In addition, DDPMs can be trained on a separate, larger dataset to generate synthetic "pretraining" data for a classifier model that is later fine-tuned on task-specific but smaller datasets, that is, to facilitate transfer learning. Finally, DDPMs might be used to conditionally generate data with specific properties to serve as null models or realistic control data for neuroscientific measures, such as those measuring neural coupling in hyperscanning experiments.[54,55]

DDPMs can also handle more complex missingness patterns. In this work, we have demonstrated the ability of DDPMs to perform cross-channel imputation, although completely missing channels represent only one pattern of missingness that can occur in real neurophysiological recordings (i.e., dead electrode). Many other patterns of missingness are possible, such as when some time points within a recorded channel are missing due to momentary artifacts. Imputation in this context would mean filling in these missing measurements given information from other channels and observed values in the channel itself. These more complex missingness patterns can be realized with diffusion models under both training strategies: for unconditional training, all possible missingness patterns work out of the box via inpainting (i.e., Figure 2E). For conditional training, the training strategy needs to be generalized from dropping out whole channels to more complex missingness patterns. Tashiro et al.[31] discuss several strategies for this purpose. A special case is the common task of time series forecasting, where the missing values are simply unobserved time points in the future. Like imputation, time series forecasting can then be realized by diffusion models trained unconditionally or conditionally.

An additional benefit of our fully convolutional denoising network is that it allows users to generate neurophysiological recordings of arbitrary length despite training on fixed-size time series. To generate long synthetic recordings, the denoising network is simply applied to the entire time series without any further technical modifications (though dependencies longer than the timescale of the training segment may not be correctly modeled). We have not used this capability here, but it will be important when generating input to a simulation where long synthetic recordings are required.

Limitations

Although diffusion models are a very popular type of deep generative models today, they are not without limitations. Because DDPMs generate samples by successively denoising them over (typically) hundreds of denoising steps, inference is computationally intensive, especially when compared to other families of deep generative models such as VAEs or GANs. Much recent work has focused on speeding up the inference time of diffusion models, often by tolerating a small degradation in sample quality.[56–58] While in this work, we applied the standard formulation

of DDPMs without further speedup, all progress in this direction is directly applicable to our setting and provides a potential remedy for the comparatively long inference times.

Furthermore, diffusion models typically operate on the dimensionality of the original data space. In neuroscience, however, there has been much interest in lower-dimensional state-space representations of high-dimensional neurophysiological recordings, especially neuronal population spiking data,[59] but more recently also continuous time series such as LFPs.[60] One way to obtain such low-dimensional neural representations with generative models is to use VAEs (e.g., like in Pandarinath et al.[13]), which model the original data distribution via a mapping to a lower-dimensional space of fixed dimensionality. This encoding property is not automatically fulfilled by DDPMs, but combinations of encoder-decoder architectures with a diffusion model in the latent space have been successfully applied to image generation and fMRI decoding.[9,25] Similar approaches are possible for neurophysiological recordings and may provide low-dimensional representations, along with increased computational efficiency. In particular, a shared latent space could be beneficial in applications similar to our imputation experiment on the AJILE12 dataset (Figure 3). Unlike the autoencoder-based model described in Talukder et al.,[30] we trained an individual DDPM for each of the twelve participants. Therefore, a potential extension of our current model is to jointly train a latent diffusion model using participant-specific encoder and decoder layers, which could increase performance and robustness due to its ability to generalize over different participants. In addition, imputation performance for the AJILE12 dataset might be improved further with more targeted variations of our architecture following the architectural principles proposed in, for example, Tashiro et al.[31] or Alcaraz and Strodthoff[32] or by applying recent diffusion-based methods to solve inverse problems.[61]

As discussed earlier, synthetic training data can alleviate privacy concerns. However, our DDPM-generated synthetic data are private in the sense that the minimum difference between time series in the real training data is similar to the minimum difference between time series in the real and generated data. While intuitive, this measure does not provide theoretical guarantees against any form of reverse engineering or memorization.[62]

Lastly, a persisting challenge in modeling neurophysiological recordings is to capture very long-range dependencies over timescales of minutes or even hours. In our training setup, the time series extracted from a long neurophysiological recording are treated as independent samples by the model. Thus, temporal dependencies beyond the length of the time series used for training are not captured. Naturally, for any given dataset, long-range and cross-scale dependencies are much harder to capture due to data sparsity. Thus, building deep generative models that are more data efficient and can capture long-range dependencies by training on only a few samples is a challenging but exciting avenue for further research.

Conclusion

Together with a flexible convolutional denoising network and the use of the OU processes, DDPMs provide a powerful and flexible type of generative model for neurophysiological recordings that can generate high-quality samples and be used in a variety of applications relevant to neuroscience like simulation, imputation, or brain-state classification.

EXPERIMENTAL PROCEDURES

Resource availability

Lead contact

Further information and requests for resources should be directed to and will be fulfilled by the lead contact Julius Vetter (julius.vetter@uni-tuebingen.de).

Materials availability

This study did not generate any new materials.

Data and code availability

- Code for our method and to reproduce our existing results is available at https://github.com/mackelab/neural_timeseries_diffusion and has been deposited at Zenodo.[63]
- All four datasets used in this work are publicly available: rat LFP,[46] https://crcns.org/data-sets/hc/hc-24; AJILE12,[45] https://dandiarchive.org/dandiset/000055/0.220127.0436; macaque ECoG,[52] http://www.neurotycho.org/anesthesia-task; and BCI Challenge @ NER 2015,[43] https://www.kaggle.com/competitions/inria-bci-challenge/.

DDPMs

To train our diffusion models, we consider a training dataset of n time windows extracted from neurophysiological recordings $\{\mathbf{x}^{(i)}\}_{i=1}^{n}$, where $\mathbf{x}^{(i)} \in \mathbb{R}^{C\times L}$ with C channels of fixed length L time steps. During sampling or inference, arbitrary time series lengths are admissible due to our fully convolutional network architecture.

Background on DDPMs

Our primary goal is to learn a parametrized probability distribution $p_\theta(\mathbf{x})$ on $\mathbb{R}^{C\times L}$ that is close to the original data distribution $p(\mathbf{x})$. $p_\theta(\mathbf{x})$ can then be used for many different tasks like imputation, generation, or likelihood evaluation. We use DDPMs.[21]

DDPMs work by modeling two processes, the reverse (denoising) and forward (noising) diffusion processes, over a sequence of latent variables $\mathbf{x}_t$, $t = 1,\ldots,T$, where T is a prespecified process length. Note that T is the number of diffusion steps, a hyperparameter in the generative model, and not the length of the time series being modeled (L above). The forward process is given by a Markov chain

$$q(\mathbf{x}_{1:T}|\mathbf{x}_0) := \prod_{t=1}^{T} q(\mathbf{x}_t|\mathbf{x}_{t-1}), \quad \text{(Equation 1)}$$

where the noising distribution

$$q(\mathbf{x}_t|\mathbf{x}_{t-1}) := \mathcal{N}\left(\mathbf{x}_t; \sqrt{1-\beta_t}\,\mathbf{x}_{t-1}, \beta_t\mathbf{I}\right) \quad \text{(Equation 2)}$$

iteratively adds Gaussian noise controlled by the noise level $\beta_t > 0$, resulting in an evolution from the data distribution, $p(\mathbf{x}_0)$, to the noise distribution, $p(\mathbf{x}_T)$, over the course of T diffusion steps. This forward process is chosen by the user beforehand and thus not learned.

As its name suggests, the reverse process reverses the forward process and is given by

$$p_\theta(\mathbf{x}_{0:T}) := p(\mathbf{x}_T)\prod_{t=1}^{T} p_\theta(\mathbf{x}_{t-1}|\mathbf{x}_t), \mathbf{x}_T \sim \mathcal{N}(0,\mathbf{I}), \quad \text{(Equation 3)}$$

$$p_\theta(\mathbf{x}_{t-1}|\mathbf{x}_t) := \mathcal{N}(\mathbf{x}_{t-1}; \mu_\theta(\mathbf{x}_t,t), \sigma_\theta(\mathbf{x}_t,t)\mathbf{I}),$$

that is, the reverse conditional distribution is also Gaussian, and its mean and variance are parametrized functions of the current diffusion step t and the noised data point $\mathbf{x}_t$ and, therefore, need to be learned. DDPMs parameterize $p_\theta(\mathbf{x}_{t-1}|\mathbf{x}_t)$ as a denoising distribution,

$$\mu_\theta(\mathbf{x}_t,t) = \frac{1}{\alpha_t}\left(\mathbf{x}_t - \frac{\beta_t}{\sqrt{1-\bar{\alpha}_t}}\epsilon_\theta(\mathbf{x}_t,t)\right), \quad \text{(Equation 4)}$$

$$\sigma_\theta(\mathbf{x}_t,t) = \left(\frac{1-\overline{\alpha}_{t-1}}{1-\overline{\alpha}_t}\sqrt{\beta_t}\right)^{\frac{1}{2}},$$

where ϵ_θ is a neural network with learnable weights θ.

In other words, the neural network $\epsilon_\theta(\mathbf{x}_t,t,\text{cond})$ acts as denoiser that, together with the diffusion time step t, and, optionally, additional conditioning information cond, produces a denoised version of the data point (in this case a time series) as the basis for the next denoising step.

The denoising network can be optimized by computing a variational lower bound on $p_\theta(\mathbf{x}_t|\mathbf{x}_{t-1})$, which results in the full (simplified) training objective:

$$\min_\theta \mathcal{L}(\theta) := \min \mathbb{E}_{\mathbf{x}_0,\epsilon}\left[\ \| \epsilon - \epsilon_\theta(\mathbf{x}_t,t)\|_2^2\ \right], \qquad \text{(Equation 5)}$$

$$\textit{with}\ \ \mathbf{x}_t = \sqrt{\overline{\alpha}_t}\mathbf{x}_0 + (1-\overline{\alpha}_t)\epsilon.$$

DDPMs for neurophysiological time series

For our application of modeling neurophysiological time series, we extend the standard DDPM setting with two modifications.

(1) First, we use a flexible convolutional network architecture based on structured convolutions[36] as our denoising network. Structured convolutions belong to a family of recent, highly expressive sequence-to-sequence models.[32,37,38] Our architecture is able to accurately model long (thousands of time points) and highly multivariate time series (over 100 channels).

(2) We sometimes replace the white noise process used in the standard DDPM with general Gaussian processes. This allows us to include desirable spectral properties into the generative process.[34] In this paper, we specifically focus on the OU process due to its advantageous numerical properties and relevance for neurophysiological recordings, particularly the inverse power law scaling over frequencies.

Network architecture with structured convolutions

Our denoising network consists of interleaved layers of structured convolutions[36] and linear layers that "mix" information between channels.[64]

Structured long convolutions are created by concatenating several linearly interpolated and exponentially downscaled convolutional kernels. The resulting convolutional kernel can be as long as the time series itself. This construction is inspired by recent advances in deep state-space models[32,37,38] and allows for long yet parameter-efficient convolutional kernels that can model complex and long-range dependencies within a given time series. To make the use of these large convolutional kernels tractable, the convolution operation is computed by element-wise multiplication in the Fourier domain after applying a fast Fourier transform. This approach drastically reduces the computation time for large kernels and long time series.

The input layer is a standard convolutional layer[65] that maps the input into a high-dimensional latent space. We parameterize the size of the latent space per channel; that is, we specify the number of latent dimensions per channel. The full dimensionality of the latent space is then given by the number of channels times the latent dimension per channel. The output layer is also a standard convolutional layer that maps the hidden activations to the desired output dimensionality. The hidden layers consist of blocks of structured convolutions followed by linear layers. The linear layers mix the output from the structured convolutions, which operate separately on each hidden channel. Unlike the original implementation of structured convolutions, we introduce the number of scales as a new hyperparameter. This means that the overall size of the structured convolutional kernel is given by $\text{kernel\_size}\cdot 2^{\text{num\_scales}-1}$.

In our experiments, especially when training on neurophysiological recordings with a high channel count, we noticed that fully parametrized linear mixing layers can cause convergence issues. The number of weights in the linear layers grows quadratically with the full latent dimensions and can quickly dwarf the number of weights in convolutional layers. To avoid this overparameterization and alleviate the convergence issues, we sparsify the weight matrices of the linear layers. To do so, we decrease the number of active weights in off-diagonal blocks, where the size of the off-diagonal blocks is a hyperparameter.

Throughout our architecture, we use Gaussian error linear unit (GELU) activation functions[66] and batch or layer normalization.[64,67,68] Importantly, our architecture does not perform any compression in time. This allows the denoising architecture to operate on time series of arbitrary length.

Alternative noise processes as diffusion process

It is straightforward to replace the white noise process commonly used in DDPMs with other Gaussian processes.[34] Instead of using white noise from $\mathcal{N}(0,I)$, the (forward) diffusion process is performed with noise sampled from a general Gaussian process $\mathcal{N}(0,\Sigma)$ with prespecified covariance Σ. The objective from Equation 5 is also changed to include Σ:

$$\min_\theta \mathcal{L}(\theta) := \min \mathbb{E}_{\mathbf{x}_0,\epsilon}\left[(\epsilon - \epsilon_\theta(\mathbf{x}_t,t))^T\Sigma^{-1}(\epsilon - \epsilon_\theta(\mathbf{x}_t,t))\right], \qquad \text{(Equation 6)}$$

where $\mathbf{x}_t = \sqrt{\overline{\alpha}_t}\mathbf{x}_0 + (1-\overline{\alpha}_t)\epsilon$ and $\epsilon \sim \mathcal{N}(0,\Sigma)$. This replacement is useful for providing the model with domain knowledge about the recordings and can lead to better empirical performance.

One such example of domain knowledge in the context of neurophysiological recordings is continuity. Neurophysiological recordings are typically continuous and do not exhibit large and abrupt jumps. In addition, for many neurophysiological recordings, the frequency spectrum exhibits inverse power-law scaling.[39]

In this paper, we use the OU process, which is a continuous noise process with a power-law frequency spectrum, to encode this domain knowledge.

Using the OU process, whose covariance kernel is given by

$$k(x_{\tau_1},x_{\tau_2}) = \sigma^2 e^{-|\tau_1-\tau_2|}, \qquad \text{(Equation 7)}$$

is computationally feasible, even for long time series. While, in general, sampling from Gaussian processes scales cubically with the length of the time series, for the OU process, it is possible in linear time. This is because the OU process can be described equivalently by the stochastic differential equation

$$dx_\tau = -\rho x_\tau d\tau + \sigma dW_\tau, \qquad \text{(Equation 8)}$$

where W_τ is the Wiener process. Furthermore, since the OU process is a stationary Gauss-Markov process, its precision matrix Σ^{-1} is banded, and the modified DDPM training objective can also be computed in linear time. For both sampling and computing the training objective, we apply the OU process independently for each time series channel with a fixed lengthscale ρ. However, we find that this modification does not improve sample quality in all datasets. See experiments on the effect of using the OU process versus white noise in the supplemental information.

Model training and inference

Our convolutional denoising networks are trained with stochastic gradient descent on the objective given in Equation 6. We train all our models using the AdamW optimizer[69] and choose hyperparameters by inspecting the quality of the generated data with respect to the training set. We check for overfitting by computing pairwise distances between real and generated data in the time and frequency domains. See the supplemental information for further details and network and training hyperparameters (Table S2). To sample and impute using the trained denoising network, a sample from the Gaussian distribution (white noise or OU) is gradually denoised following the standard DDPM denoising procedure.[21]

DDPMs naturally lend themselves to imputation by guiding the diffusion process based on observed data points.[21] For image-based diffusion models, this process is usually known as inpainting.[49] It works by setting the observed part of the image—or time series—to the analytical latent state of the forward diffusion process (as given by Equation 1) after each denoising step. This way, the reverse diffusion process is "guided" along the observed part of the time series. However, this approach does not provide the correct conditional distributions of the form $p(\mathbf{x}_{\text{observed}}|\mathbf{x}_{\text{missing}})$, which can hurt imputation performance.[31]

To alleviate this issue, it is possible to train conditional diffusion models: here, the observed data are given as input to the denoising network; that is, the diffusion model is trained to model the desired conditional distribution directly. In order to obtain a conditional diffusion model that can deal with arbitrary patterns of missingness at imputation time, many different possible such

patterns need to be sampled during training. In this work, we use the random sampling strategy[31] and randomly drop out channels for a given time series following a two-step process: first, we uniformly sample a number k between one and the total number of channels C and then randomly drop out k channels. The denoising network is then conditioned on the remaining $C - k$ channels, and the training objective in Equation 6 is computed. We use this conditional training strategy only for the AJILE12 dataset. For the other two datasets, the models are trained without randomly dropping out channels. All models are implemented and trained with the PyTorch library.[70]

Datasets

We perform our three experiments on three different datasets of publicly available neurophysiological recordings.

The dataset for our first experiment (Figure 2) is a three-electrode LFP recording from rats recorded at 600 Hz at the mPFC, hippocampal CA1 region, and thalamic nucleus RE.[44] It consists of a total of 3.5 h of recordings from three different rats. During the recording, rats were freely behaving with the possibility to rest and underwent cycles of sleep and wakefulness. For our experiment, we used the LFP data from the first and third recordings (52 and 93 min).

The AJILE12 dataset used for our second experiment (Figure 3) consists of human ECoG data recorded during naturalistic behavior from 12 different participants undergoing epilepsy monitoring.[45] It was recorded at 500 Hz over several days and is a total of 1,280 h. The length of recording per participant ranges from 70 to 120 h. The number of electrodes per participant ranges from 64 to 126. In addition to the neurophysiological recordings, a variety of behavioral- and movement-event-related metadata are provided.

The dataset for our third experiment (Figure 4) consists of ECoG recordings from a nonhuman primate (macaque monkey) recorded at 1,000 Hz via 128 surface electrodes placed inside the cranium, directly on top of the cortex.[52] During the recording, the macaque (Chibi) was injected with an anesthetic (propofol), and different neurophysiological states were annotated. We use the data from the anesthetized and awake-eyes-closed condition (73 min of recording).

For our initial example (Figure 1E–1H), we use data from participant P02 of the BCI Challenge @ NER 2015.[43] The data were recorded at 200 Hz with 56 passive EEG sensors placed with the extended 10–20 system. The participants were tested under the "P-300 Speller" paradigm, where words are spelled out letter by letter by flashing screen items and measuring the evoked response.[71]

Data preprocessing

For our datasets of rat LFP and macaque ECoG recordings, we perform channel-by-channel normalization by standardizing the recording over its entire duration. After normalization, we create the set of time windows that are used to train and evaluate the model. For the rat LFP recordings, we took 2-s time windows consisting of 1,200 time points, resulting in a total of 4,350 time series. Similarly, for the macaque ECoG dataset, we took 1-s time windows consisting of 1,000 time points, resulting in a total of 1,088 time series. After creating the time series, we then randomly divide them into a training and an evaluation set (80% training, 20% evaluation).

The data of each participant in the AJILE12 dataset consist of 7 days of recording. We follow the setup and preprocessing described in Peterson et al.[51]: the ECoG traces are downsampled before extracting time windows corresponding to either movement or rest. For each participant, the final dataset consists of 4-s time windows with a sampling frequency of 250 Hz, with an equal amount of time series recorded under rest and movement (i.e., class balanced). Again, we normalize the time series channel by channel by aggregating over all time series in the training set. The normalization of the training set, which consists of the first 6 days of recording, is applied to the evaluation set, that is, the seventh day of recording. Furthermore, extreme outlier channels, where the difference between the minimum and maximum values is five or more times the interquartile range, are detected and masked during training and evaluation. The number of recorded channels varies widely between participants, ranging from 64 to 126. Similarly, the number of training and evaluation time series varies considerably between participants. However, the network hyperparameters are shared between participants, and no attempt was made to fine-tune the imputation for an individual participant.

Unlike the DDPM, the random forest neural decoder is trained and evaluated on the unnormalized AJILE12 data (see Peterson et al.[51] for details). Imputations are obtained by imputing normalized time series and then reversing the normalization for both the observed and imputed channels using the normalization constants from the training data.

For our EEG dataset, we extract 1.3 s of the recording immediately following a presented stimulus. Since we only use data from participant P02, this results in 340 time series (i.e., trials) consisting of 260 time points each. After extraction, we band-pass filter the time series with a frequency window between 1 and 40 Hz using a fifth-order Butterworth filter. We then again perform channel-by-channel normalization by aggregating over the time series.

Analyses and metrics

PSDs

Throughout the experiments, we compare the distributions of (cross-)PSDs between channels of real and generated data. PSDs are computed with a Fourier transform over the whole the time series. For a given one-dimensional time series x and frequency f, the PSD is denoted by $S_{xx}(f)$. Since we are always interested in capturing the full distribution of PSDs over a set of time series, we compute the point-wise median and percentiles: here, point-wise means that, for a set of real or generated time series $\{\mathbf{x}_i\}_{i=1}^{n}$, the median $\text{median}[S_{xx}(f)]$ over all PSDs is computed at each frequency f, and similarly for the 10% and 90% percentiles.

SWR detection and cross-channel couplings

For our experiments with the rat LFP data, we detect ripples in the CA1 channel. Following the heuristic approach of Varela and Wilson,[44] we perform this detection by band-pass filtering both real and generated CA1 traces within the ripple frequency band from 100 to 275 Hz using a finite impulse response (FIR) filter. Ripples are detected when the power of the time series is more than three standard deviations greater than the mean. The mean and standard deviation of the filtered power are calculated over the entire set of real time series. Crucially, to ensure a fair comparison between a set of real and a set of generated CA1 traces, the mean and standard deviation from the real data are used to detect SWRs in the generated data. SWRs with less than 10 time points (17 ms) are fused. After fusing, an SWR must have a minimum length of 20 time points (33 ms). Detected ripples below this threshold are discarded.

In our SWR prediction experiment, we impute the CA1 channel given the other two channels and detect SWRs in the real and imputed channel for all time series in the evaluation set. If there are no SWRs or at least one SWR in both the real and generated channels, then the prediction is counted as correct. We then compute the F1 score based on the number of correct and incorrect predictions. To test if the prediction performance is significantly better than random, we shuffle the set of generated time series and evaluate the F1 score. This procedure is repeated 1,000 times to compute the permutation test statistic.[50]

We also use detected SWRs to compute a "phase-occurrence coupling" against the phase of delta (1–4 Hz), theta (4–9 Hz), and spindle (6–14 Hz) frequencies in the mPFC. The mPFC phase is calculated using the Hilbert transform after band-pass filtering (FIR, Hamming) with the corresponding frequency band. Given the mPFC phase, the time point with maximal power within a detected SWR is used to extract the phase of the SWR occurrence, and the counts are binned into 21 equally spaced phase bins (from -180° to 180°) to create the histograms (Figure 2D).

Finally, we compute the PAC between the CA1 amplitude in the ripple band (100–275 Hz) and the mPFC phase of the previously mentioned driver frequencies. Phase and amplitude in the respective frequency bands are calculated using the Hilbert transform, and the average amplitudes occurring at 31 equally spaced phase bins (from -180° to 180°) are reported (Figure 2D).

Details on the imputation experiment and neural decoder

For our imputation experiment, we follow the setup described in Talukder et al.[30]: we impute artificially dropped channels and investigate the effect of this imputation on the performance of a neural decoder. As a baseline imputation method, we use mean imputation. The neural decoding task is to discriminate between time series recorded at rest and time series recorded during movement. The neural decoder used is a random forest, which works by "flattening" a time series into a large feature vector. We train three decoders over three training folds as in Peterson et al.[51] using the same hyperparameters (maximum tree depth and number of estimators). No attempt was made to

further optimize the random forest models. Finally, the average test performance over all training folds is calculated for all settings, that is, fully observed, mean imputation, deterministic-network-based imputation, and DDPM-based imputation.

To evaluate the performance of DDPM-based imputation, we randomly select 50%, 70%, or 90% of the channels from a given participant. For each time series in the evaluation set, this set of channels is dropped and then imputed with the mean from the training set or imputed using the trained DDPM. We then apply the previously trained decoder to both imputed evaluation sets and compute the evaluation accuracy. This process is repeated 5 times for each missingness level to evaluate the imputation performance over different sets of dropped channels. We report the mean and standard deviation over the 5 repetitions (Figure 3).

Likelihood computation and brain-state classification

Likelihood computation in DDPMs can be realized by solving the probability flow equation.[72] In our class-conditional model for the macaque ECoG data, we compute log likelihoods for both the awake and anesthetized states. That is, given a time series $\mathbf{x}$, we get $\log p_\theta(\mathbf{x}|c = \text{awake})$ and $\log p_\theta(\mathbf{x}|c = \text{anesthetized})$. These values can be used to perform classification by selecting the class with the higher log likelihood for a given sample. We perform this classification for all time series in our class-balanced evaluation set and report the overall classification accuracy. For the time-resolved brain state classification (Figure 4E), we simply compute this log likelihood difference over 1-s sliding windows.

For outlier detection, we are interested in the total log likelihood $\log p_\theta(\mathbf{x})$ of sample $\mathbf{x}$. We compute this total log likelihood for each evaluation time series and correspondingly generated outliers (white noise or time-wise flipped) according to the law of total probability. Then, the AUC of the time series against both types of outliers is computed.

For the brain-state classification with synthetic data, we use the convolutional neural network classifier described in Wang et al.[53] The classifier is trained twice, once on real data and once on an equal amount of DDPM-generated data, and then evaluated on a held-out evaluation set of real data. Both the training and evaluation datasets consist of an equal number of awake and anesthetized time windows, and we compute the evaluation accuracy to compare the performance between the real and synthetic settings. The classifier is trained using the AdamW optimizer[69] and binary cross-entropy loss with identical network and training hyperparameters in both settings.

SUPPLEMENTAL INFORMATION

Supplemental information can be found online at https://doi.org/10.1016/j.patter.2024.101047.

ACKNOWLEDGMENTS

R.G. was supported by the European Union's Horizon 2020 research and innovation programme under the Marie Skłodowska-Curie grant agreement no. 101030918 (AutoMIND). This work was supported by the German Research Foundation (DFG) through Germany's Excellence Strategy, EXC number 2064/1–390727645 and SFB1233 (PN 276693517); the German Federal Ministry of Education and Research (BMBF): Tübingen AI Center, FKZ: 01IS18039A; and the European Union (ERC, DeepCoMechTome, 101089288). J.V. is a member of the International Max Planck Research School for Intelligent Systems (IMPRS-IS) and the AI4Med-BW graduate program. We would like to thank Auguste Schulz and Matthijs Pals for feedback on the manuscript and Mackelab members for discussions.

AUTHOR CONTRIBUTIONS

Conceptualization, J.V., J.H.M., and R.G.; formal analysis, J.V.; methodology, J.V.; visualization, J.V.; software, J.V.; supervision, J.H.M. and R.G.; writing – original draft, J.V.; writing – review & editing, J.V., J.H.M., and R.G.; funding acquisition, J.H.M.

DECLARATION OF INTERESTS

The authors declare no competing interests.

Received: April 6, 2024
Revised: July 1, 2024
Accepted: July 31, 2024
Published: August 29, 2024

REFERENCES

1. Krumin, M., and Shoham, S. (2009). Generation of spike trains with controlled auto-and cross-correlation functions. Neural Comput. *21*, 1642–1664.
2. Macke, J.H., Berens, P., Ecker, A.S., Tolias, A.S., and Bethge, M. (2009). Generating spike trains with specified correlation coefficients. Neural Comput. *21*, 397–423.
3. Gutnisky, D.A., and Josić, K. (2010). Generation of spatiotemporally correlated spike trains and local field potentials using a multivariate autoregressive process. J. Neurophysiol. *103*, 2912–2930.
4. Schreiber, T., and Schmitz, A. (2000). Surrogate time series. Phys. Nonlinear Phenom. *142*, 346–382.
5. Venema, V., Ament, F., and Simmer, C. (2006). A stochastic iterative amplitude adjusted Fourier transform algorithm with improved accuracy. Nonlinear Process Geophys. *13*, 321–328.
6. Gifford, A.T., Dwivedi, K., Roig, G., and Cichy, R.M. (2022). A large and rich EEG dataset for modeling human visual object recognition. Neuroimage *264*, 119754.
7. Kong, Z., Ping, W., Huang, J., Zhao, K., and Catanzaro, B. (2021). Diffwave: A versatile diffusion model for audio synthesis. International Conference on Learning Representations *abs/2009.09761*. https://doi.org/10.48550/arXiv.2009.09761.
8. Dhariwal, P., and Nichol, A. (2021). Diffusion models beat GANs on image synthesis. Adv. Neural Inf. Process. Syst. *34*, 8780–8794.
9. Rombach, R., Blattmann, A., Lorenz, D., Esser, P., and Ommer, B. (2022). High-resolution image synthesis with latent diffusion models. Proceedings of the IEEE/CVF Conference on Computer Vision and Pattern Recognition, 10684–10695.
10. Fortuin, V., Baranchuk, D., Rätsch, G., and Mandt, S. (2020). GP-VAE: Deep probabilistic time series imputation. International Conference on Artificial Intelligence and Statistics, 1651–1661. https://doi.org/10.48550/arXiv.1907.04155.
11. Sanchez-Lengeling, B., and Aspuru-Guzik, A. (2018). Inverse molecular design using machine learning: Generative models for matter engineering. Science *361*, 360–365.
12. Sun, H., and Bouman, K.L. (2021). Deep probabilistic imaging: Uncertainty quantification and multi-modal solution characterization for computational imaging. Proc. AAAI Conf. Artif. Intell. *35*, 2628–2637.
13. Pandarinath, C., O'Shea, D.J., Collins, J., Jozefowicz, R., Stavisky, S.D., Kao, J.C., Trautmann, E.M., Kaufman, M.T., Ryu, S.I., Hochberg, L.R., et al. (2018). Inferring single-trial neural population dynamics using sequential auto-encoders. Nat. Methods *15*, 805–815.
14. Molano-Mazon, M., Onken, A., Piasini, E., and Panzeri, S. (2018). Synthesizing realistic neural population activity patterns using generative adversarial networks. International Conference on Learning Representations. https://doi.org/10.48550/arXiv.1803.00338.
15. Ramesh, P., Atayi, M., and Macke, J.H. (2019). Adversarial training of neural encoding models on population spike trains. Real Neurons & Hidden Units: Future directions at the intersection of neuroscience and artificial intelligence @ NeurIPS 2019.
16. Aznan, N.K.N., Atapour-Abarghouei, A., Bonner, S., Connolly, J.D., Al Moubayed, N., and Breckon, T.P. (2019). Simulating brain signals: Creating synthetic eeg data via neural-based generative models for improved ssvep classification. In 2019 International joint conference on neural networks (IJCNN), pp. 1–8.
17. Hartmann, K.G., Schirrmeister, R.T., and Ball, T. (2018). Eeg-gan: Generative adversarial networks for electroencephalograhic (eeg) brain signals. Preprint at arXiv. https://doi.org/10.48550/arXiv.1806.01875.

18. Luo, Y., Zhu, L.-Z., Wan, Z.-Y., and Lu, B.-L. (2020). Data augmentation for enhancing eeg-based emotion recognition with deep generative models. J. Neural. Eng. *17*, 056021.

19. Ponce, C.R., Xiao, W., Schade, P.F., Hartmann, T.S., Kreiman, G., and Livingstone, M.S. (2019). Evolving images for visual neurons using a deep generative network reveals coding principles and neuronal preferences. Cell *177*, 999–1009.e10.

20. Lin, S., Sprague, T., and Singh, A.K. (2022). Mind reader: Reconstructing complex images from brain activities. Adv. Neural Inf. Process. Syst. *35*, 29624–29636.

21. Ho, J., Jain, A., and Abbeel, P. (2020). Denoising diffusion probabilistic models. Adv. Neural Inf. Process. Syst. *33*, 6840–6851.

22. Yang, L., Zhang, Z., Song, Y., Hong, S., Xu, R., Zhao, Y., Zhang, W., Cui, B., and Yang, M.-H. (2023). Diffusion models: A comprehensive survey of methods and applications. ACM Comput. Surv. *56*, 1–39.

23. Lin, L., Li, Z., Li, R., Li, X., and Gao, J. (2024). Diffusion models for time series applications: A survey. Front. Inform. Technol. Electron. Eng. *25*, 19–41.

24. Takagi, Y., and Nishimoto, S. (2023). High-resolution image reconstruction with latent diffusion models from human brain activity. Proceedings of the IEEE/CVF Conference on Computer Vision and Pattern Recognition, 14453–14463. https://doi.org/10.1101/2022.11.18.517004.

25. Chen, Z., Qing, J., Xiang, T., Yue, W.L., and Zhou, J.H. (2023). Seeing beyond the brain: Conditional diffusion model with sparse masked modeling for vision decoding. Proceedings of the IEEE/CVF Conference on Computer Vision and Pattern Recognition, 22710–22720. https://doi.org/10.48550/arXiv.2211.06956.

26. Silva, I., Moody, G., Scott, D.J., Celi, L.A., and Mark, R.G. (2012). Predicting in-hospital mortality of icu patients: The physionet/computing in cardiology challenge 2012. Comput. Cardiol. *39*, 245–248.

27. Walonoski, J., Kramer, M., Nichols, J., Quina, A., Moesel, C., Hall, D., Duffett, C., Dube, K., Gallagher, T., and McLachlan, S. (2018). Synthea: An approach, method, and software mechanism for generating synthetic patients and the synthetic electronic health care record. J. Am. Med. Inform. Assoc. *25*, 230–238.

28. Yoon, J., Jarrett, D., and Van der Schaar, M. (2019). Time-series generative adversarial networks. Adv. Neural Inf. Process. Syst. *32*, 5509–5519.

29. Abbasi, S., Maran, S., and Jaeger, D. (2020). A general method to generate artificial spike train populations matching recorded neurons. J. Comput. Neurosci. *48*, 47–63.

30. Talukder, S., Sun, J.J., Leonard, M., Brunton, B.W., and Yue, Y. (2022). Deep neural imputation: A framework for recovering incomplete brain recordings. NeurIPS 2022 Workshop on Learning from Time Series for Health. https://doi.org/10.48550/arXiv.2206.08094.

31. Tashiro, Y., Song, J., Song, Y., and Ermon, S. (2021). CSDI: Conditional score-based diffusion models for probabilistic time series imputation. Adv. Neural Inf. Process. Syst. *34*, 24804–24816.

32. Alcaraz, J.L., and Strodthoff, N. (2022). Diffusion-based time series imputation and forecasting with structured state space models. Transactions on Machine Learning Research. https://doi.org/10.48550/arXiv.2208.09399.

33. Rasul, K., Seward, C., Schuster, I., and Vollgraf, R. (2021). Autoregressive denoising diffusion models for multivariate probabilistic time series forecasting. International Conference on Machine Learning, 8857–8868. https://doi.org/10.48550/arXiv.2101.12072.

34. Biloš, M., Rasul, K., Schneider, A., Nevmyvaka, Y., and Günnemann, S. (2023). Modeling temporal data as continuous functions with process diffusion. International Conference on Machine Learning *202*, 2452–2470.

35. Shu, K., Zhao, Y., Wu, L., Liu, A., Qian, R., and Chen, X. (2023). Data augmentation for seizure prediction with generative diffusion model. Preprint at arXiv. https://doi.org/10.48550/arXiv.2306.08256.

36. Li, Y., Cai, T., Zhang, Y., Chen, D., and Dey, D. (2023). What makes convolutional models great on long sequence modeling? International Conference on Learning Representations *abs/2210.09298*. https://doi.org/10.48550/arXiv.2210.09298.

37. Gu, A., Goel, K., and Ré, C. (2022). Efficiently modeling long sequences with structured state spaces. International Conference on Learning Representations *abs/2111.00396*. https://doi.org/10.48550/arXiv.2111.00396.

38. Smith, J.T., Warrington, A., and Linderman, S.W. (2023). Simplified state space layers for sequence modeling. International Conference on Learning Representations *abs/2208.04933*. https://doi.org/10.48550/arXiv.2208.04933.

39. Pritchard, W.S. (1992). The brain in fractal time: 1/f-like power spectrum scaling of the human electroencephalogram. Int. J. Neurosci. *66*, 119–129.

40. He, B.J., Zempel, J.M., Snyder, A.Z., and Raichle, M.E. (2010). The temporal structures and functional significance of scale-free brain activity. Neuron *66*, 353–369.

41. Gao, R., Peterson, E.J., and Voytek, B. (2017). Inferring synaptic excitation/inhibition balance from field potentials. Neuroimage *158*, 70–78.

42. Donoghue, T., Haller, M., Peterson, E.J., Varma, P., Sebastian, P., Gao, R., Noto, T., Lara, A.H., Wallis, J.D., Knight, R.T., et al. (2020). Parameterizing neural power spectra into periodic and aperiodic components. Nat. Neurosci. *23*, 1655–1665.

43. Margaux, P., Emmanuel, M., Sébastien, D., Olivier, B., and Jérémie, M. (2012). Objective and subjective evaluation of online error correction during P300-based spelling. Advances in Human-Computer Interaction *2012*, 578295. https://doi.org/10.1155/2012/578295.

44. Varela, C., and Wilson, M.A. (2020). mPFC spindle cycles organize sparse thalamic activation and recently active CA1 cells during non-REM sleep. Elife *9*, e48881.

45. Peterson, S.M., Singh, S.H., Dichter, B., Scheid, M., Rao, R.P.N., and Brunton, B.W. (2022). AJILE12: Long-term naturalistic human intracranial neural recordings and pose. Sci. Data *9*, 184.

46. Varela, C., and Wilson, M.A. (2019). Simultaneous extracellular recordings from midline thalamic nuclei, medial prefrontal cortex and CA1 from rats cycling through bouts of sleep and wakefulness. CRCNS.org. https://doi.org/10.6080/K0K35RVG.

47. Kingma, D.P., and Welling, M. (2013). Auto-encoding variational bayes. International Conference on Learning Representations. https://doi.org/10.48550/arXiv.1312.6114.

48. Goodfellow, I., Pouget-Abadie, J., Mirza, M., Xu, B., Warde-Farley, D., Ozair, S., Courville, A., and Bengio, Y. (2020). Generative adversarial networks. Commun. ACM *63*, 139–144.

49. Lugmayr, A., Danelljan, M., Romero, A., Yu, F., Timofte, R., and Van Gool, L. (2022). Repaint: Inpainting using denoising diffusion probabilistic models. Proceedings of the IEEE/CVF Conference on Computer Vision and Pattern Recognition, 11461–11471.

50. Ojala, M., and Garriga, G.C. (2010). Permutation tests for studying classifier performance. J. Mach. Learn. Res. *11*.

51. Peterson, S.M., Steine-Hanson, Z., Davis, N., Rao, R.P.N., and Brunton, B.W. (2021). Generalized neural decoders for transfer learning across participants and recording modalities. J. Neural. Eng. *18*, 026014.

52. Yanagawa, T., Chao, Z.C., Hasegawa, N., and Fujii, N. (2013). Large-scale information flow in conscious and unconscious states: An ECoG study in monkeys. PLoS One *8*, e80845.

53. Wang, Z., Yan, W., and Oates, T. (2017). Time series classification from scratch with deep neural networks: A strong baseline. International joint conference on neural networks (IJCNN), 1578–1585. https://doi.org/10.48550/arXiv.1611.06455.

54. Xu, X., Kong, Q., Zhang, D., and Zhang, Y. (2024). An evaluation of inter-brain eeg coupling methods in hyperscanning studies. Cogn. Neurodyn. *18*, 67–83.

55. Burgess, A.P. (2013). On the interpretation of synchronization in eeg hyperscanning studies: a cautionary note. Front. Hum. Neurosci. *7*, 881.

56. Song, J., Meng, C., and Ermon, S. (2021a). Denoising diffusion implicit models. International Conference on Learning Representations *abs/2010.02502*. https://doi.org/10.48550/arXiv.2010.02502.

57. Karras, T., Aittala, M., Aila, T., and Laine, S. (2022). Elucidating the design space of diffusion-based generative models. Adv. Neural Inf. Process. Syst. *35*, 26565–26577.

58. Lu, C., Zhou, Y., Bao, F., Chen, J., Li, C., and Zhu, J. (2022). DPM-Solver: A fast ODE solver for diffusion probabilistic model sampling in around 10 steps. Adv. Neural Inf. Process. Syst. *35*, 5775–5787.

59. Vyas, S., Golub, M.D., Sussillo, D., and Shenoy, K.V. (2020). Computation through neural population dynamics. Annu. Rev. Neurosci. *43*, 249–275.

60. Gallego-Carracedo, C., Perich, M.G., Chowdhury, R.H., Miller, L.E., and Gallego, J.Á. (2022). Local field potentials reflect cortical population dynamics in a region-specific and frequency-dependent manner. Elife *11*, e73155.

61. Chung, H., Kim, J., Mccann, M.T., Klasky, M.L., and Ye, J.C. (2023). Diffusion posterior sampling for general noisy inverse problems. International Conference on Learning Representations. https://doi.org/10.48550/arXiv.2209.14687.

62. van den Burg, G., and Williams, C. (2021). On memorization in probabilistic deep generative models. Adv. Neural Inf. Process. Syst. *34*, 27916–27928.

63. Vetter, J., Macke, J.H., and Gao, R. (2024). Code for the Article “Generating Realistic Neurophysiological Time Series with Denoising Diffusion Probabilistic Models (Zenodo). https://doi.org/10.5281/zenodo.12759366.

64. Peebles, W., and Xie, S. (2023). Scalable diffusion models with transformers. In Proceedings of the IEEE/CVF International Conference on Computer Vision, pp. 4195–4205. https://doi.org/10.48550/arXiv.2212.09748.

65. LeCun, Y., Bengio, Y., et al. (1995). Convolutional networks for images, speech, and time series. The handbook of brain theory and neural networks *3361*, 255–258.

66. Hendrycks, D., and Gimpel, K. (2016). Gaussian error linear units. Preprint at arXiv. https://doi.org/10.48550/arXiv.1606.08415.

67. Ioffe, S., and Szegedy, C. (2015). Batch normalization: Accelerating deep network training by reducing internal covariate shift. International Conference on Machine Learning, 448–456. https://doi.org/10.48550/arXiv.1502.03167.

68. Ba, J.L., Kiros, J.R., and Hinton, G.E. (2016). Layer normalization. Preprint at arXiv:1607.06450. https://doi.org/10.48550/arXiv.1607.06450.

69. Loshchilov, I., and Hutter, F. (2019). Decoupled weight decay regularization. International Conference on Learning Representations. https://doi.org/10.48550/arXiv.1711.05101.

70. Paszke, A., Gross, S., Massa, F., Lerer, A., Bradbury, J., Chanan, G., Killeen, T., Lin, Z., Gimelshein, N., Antiga, L., et al. (2019). Pytorch: An imperative style, high-performance deep learning library. Adv. Neural Inf. Process. Syst. *32*.

71. Farwell, L.A., and Donchin, E. (1988). Talking off the top of your head: Toward a mental prosthesis utilizing event-related brain potentials. Electroencephalogr. Clin. Neurophysiol. *70*, 510–523.

72. Song, Y., Sohl-Dickstein, J., Kingma, D.P., Kumar, A., Ermon, S., and Poole, B. (2021b). Score-based generative modeling through stochastic differential equations. International Conference on Learning Representations. https://doi.org/10.48550/arXiv.2011.13456.

Patterns, Volume *5*

Supplemental information

Generating realistic neurophysiological time series with denoising diffusion probabilistic models

Julius Vetter, Jakob H. Macke, and Richard Gao

Supplemental Information

Supplemental Experimental Procedures

Ornstein-Uhlenbeck process as diffusion process

In our experiments, we sometimes use the OU process instead of the classically used white noise as our diffusion process. This substitution is possible because the OU process belongs to the family of Gaussian processes. See Biloš et al.[1] for more details.

The training and sampling schemes are shown in Algorithm 1 and Algorithm 2 and differ slightly from those given in Biloš et al.[1]. Note that the extension to general Gaussian processes has a computational overhead compared to the original training objective. During training, the computation of the Mahalanobis distance scales quadratically with the length of the input. Sampling from a Gaussian process also requires computing a Cholesky decomposition. However, this decomposition only needs to be computed once, and can also be used to compute the Mahalanobis efficiently by solving a lower triangular linear system based on the Cholesky decomposition.

Algorithm 1 Training

1: $L = \mathrm{Cholesky}(\Sigma)$
2: **repeat**
3: $\mathbf{x}_0 \sim q(\mathbf{x}_0)$
4: $t \sim \mathrm{Uniform}(\{1, \ldots, T\})$
5: $\boldsymbol{\epsilon} \sim \mathcal{N}(0, \Sigma)$
6: Take gradient descent step on
$\nabla_\theta (\boldsymbol{\epsilon} - \boldsymbol{\epsilon}_\theta(\mathbf{x}_t, t))^T \Sigma^{-1} (\boldsymbol{\epsilon} - \boldsymbol{\epsilon}_\theta(\mathbf{x}_t, t))$
7: **until** converged

Algorithm 2 Sampling

1: $\mathbf{x}_T \sim \mathcal{N}(0, \Sigma)$
2: **for** $t = T, \ldots, 1$ **do**
3: $z \sim \mathcal{N}(0, \Sigma)$ if $t > 1$, else $z = 0$
4: $\mathbf{x}_{t-1} = \frac{1}{\sqrt{\alpha_t}}(\mathbf{x}_t - \frac{1-\alpha_t}{\sqrt{1-\bar{\alpha}_t}} \boldsymbol{\epsilon}_\theta(\mathbf{x}_t, t)) + \sigma_t z$
5: **end for**
6: **return** $\mathbf{x}_0$

As described in the main text, the computational requirements scale more favorably for the special case of the OU process. Its formulation as a stochastic differential equation allows sampling in linear time. Furthermore, the OU process has a tridiagonal precision matrix, which allows computing the training objective in linear time as well.

Additional results and details

Here we provide additional results and details. The hyperparameters used for our experiments are given in Table S2.

For completeness, all distributions of PSDs of AJILE12 participant P07 in the main text are given (Fig. S7). Additionally, all distributions of PSDs for two other participants, participant P01 with 94 channels (Fig. S8) and participant P12 with 126 channels (Fig. S9) are provided. In both cases, the median spectra as well as 10 % and 90 % percentiles match well. Recall that the neural decoding performance on data imputed by our model did not improve over the mean imputation baseline for participant P01. Nevertheless, the overall spectra of the real and imputed data match. This means that while the model was able to capture the overall distribution of time series, it likely failed to learn the class-conditional mapping between observed and missing channels. The average correlations between ground truth and imputations are positive for all 12 participants. With increasing levels of dropout, they drop towards zero (Table S1). The relative improvements of the DDPM-based imputations over the mean imputation baseline are on average better than the ones obtained by the deterministic CNNAE model (Talukder et al.[2], Table S1). We also provide an example of a deterministic, neural network-based imputation (Fig. S5).

As an additional experiment with high channel count, we generated time series from the awake condition of the macaque ECoG data on all 128 recorded channels. The distribution of real and generated PSDs closely match across all channels, but our model sometimes overestimates the power in the low-frequency region (Fig. S6).

Distances between real and generated data For all datasets, we generate the same number of samples as were used to train the corresponding models. We then compute all nearest-neighbor distances within the training data and from the generated samples to the training samples. Two distance measures are used, the L_1 distance in

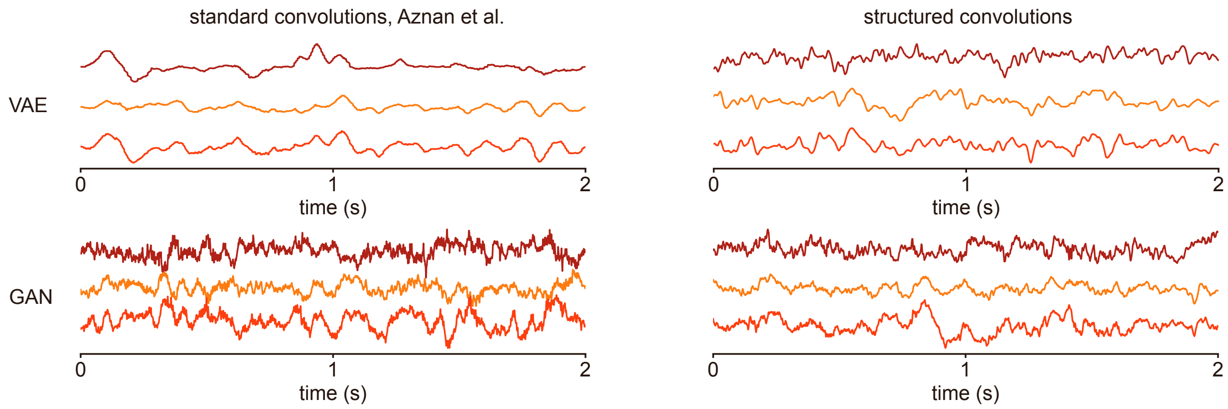

Figure S1: **Samples from GANs and VAEs with architectures based on standard and structured convolutions** The encoder/decoder and generator/discriminator architectures based on structured convolutions produce better-looking samples.

the time domain (which is small if and only if two time series are nearly exact copies of each other) and the Fourier-Wasserstein distance[3]. The Fourier-Wasserstein distance, a pseudo-metric that operates in the spectral domain, is based on normalized PSDs and will be small if, for example, two time series are scaled or translated versions of each other.

We then compare the distribution of nearest neighbor distances between the real and generated data with the distribution of distances within the training data (Fig. S4). Crucially, the minimum nearest-neighbor distance between and within the real and generated time series is away from zero for both the L_1 and the Fourier-Wasserstein distance and similar to the distribution of distances within. For a heavily overfitted DDPM, we would expect large parts of the distribution of nearest-neighbor distances between the real and generated data to be substantially smaller.

OU process versus white noise Our use of the OU process provides a modification that can further improve the quality of generated samples in some cases. Here, we provide an analysis for the AJILE12 dataset. We retrain the models using the standard independent Gaussian process (white noise). All other training and network hyperparameters are kept fixed.

For all AJILE12 participants (Fig. S10, Fig. S11, Fig. S12 for AJILE12 participants P01, P07, P12, respectively), the median power-spectra are worse using the white noise instead of the OU process, especially in the low-power, high-frequency region, which is overestimated when white noise is used.

However, for the rat LFP, the awake/anesthetized macaque ECoG data, and the BCI EEG data, there was no substantial advantage to using the OU process over white noise.

VAE and GAN baseline Here, we use the unconditional rat LFP generation experiment to establish a baseline using other types of generative models. We train VAEs and GANs using the architectures from Aznan et al.[4] as well as our own encoder/decoder and generator/discriminator architectures based on structured convolutions. The convolution hyperparameters were informed by those used in DDPM, but further tuned for the generative model at hand.

We find that the architectures used in Aznan et al.[4] are not sufficient to faithfully capture the statistics of the LFP recordings. Our architectures based on structured convolutions produce more realistic samples for both VAE and GAN (Fig. S1). However, and as we discuss below, they still fall short of the performance achieved by the DDPM:

The VAE with the encoder/decoder based on structured convolutions produces realistic but too smooth LFPs (Fig. S2A). The marginals between real and generated recordings closely match (Fig. S2B). At low frequencies, the VAE-generated samples capture the spectrum of the recordings well. At higher frequencies, however, their power drops off too quickly (Fig. S2C). As a result, the generated samples appear smooth or blurred. This is a common problem with VAEs[5,6]. Because of their smoothness, the samples do not have the correct phase-amplitude coupling (Fig. S2D).

The GAN with the generator/discriminator based on structured convolutions is able to produce realistic looking LFP recordings (Fig. S2A) whose power spectra and marginals match (Fig. S2B and C). Furthermore, we observe a non-trivial phase-amplitude coupling in the generated samples (Fig. S2D). Overall, however, the quality of the generated samples is inferior to that of the DDPM: The median spectra show strong oscillations at higher frequencies, and the couplings only approximately match those of the real data. Furthermore, we found the training of the GAN

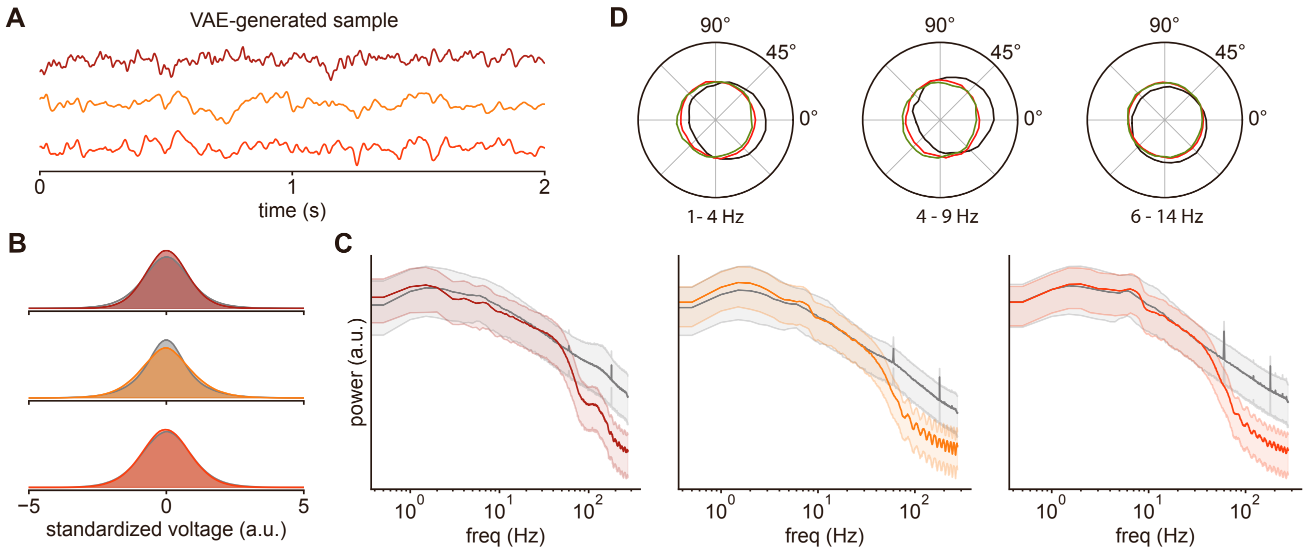

Figure S2: **VAE with structured convolutions for rat cortical-hippocampal LFP. (A)** Example of time series generated by the VAE. **(B)** Marginal distributions over the standardized voltage of each channel for both real and generated data. **(C)** Median and 10 %/90 % percentiles of real and generated power spectra for each channel. **(D)** Phase-amplitude and phase-count coupling of CA1 ripples for different driver frequencies in the mPFC for real, surrogate, and VAE-generated data.

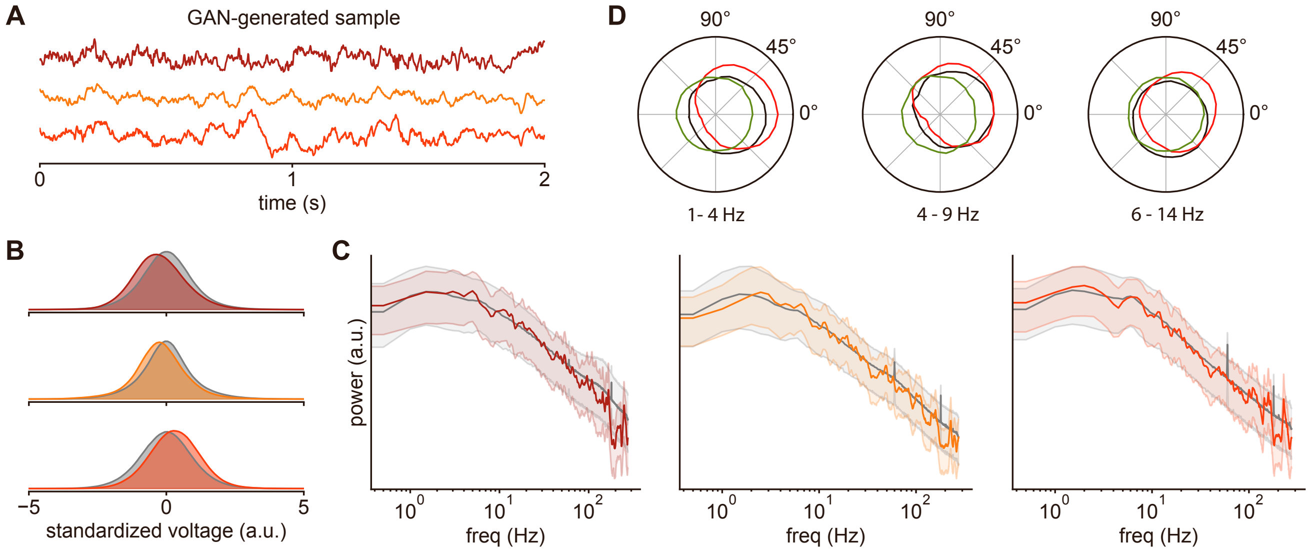

Figure S3: **GAN with structured convolutions for rat cortical-hippocampal LFP. (A)** Example of time series generated by the GAN. **(B)** Marginal distributions over the standardized voltage of each channel for both real and generated data. **(C)** Median and 10 %/90 % percentiles of real and generated power spectra for each channel. **(D)** Phase-amplitude and phase-count coupling of CA1 ripples for different driver frequencies in the mPFC for real, surrogate, and GAN-generated data.

to be relatively unstable[7,8]. Different seeds can lead to very different results, even if the chosen hyperparameters are kept fixed.

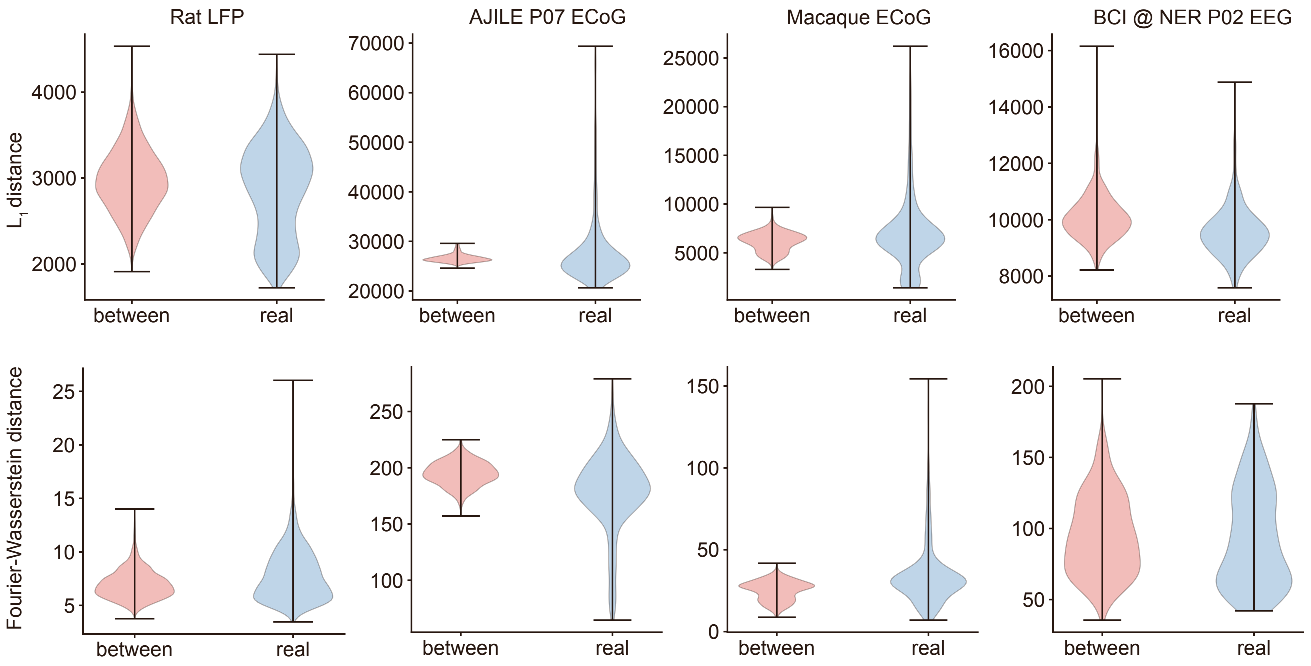

Figure S4: **Distribution of nearest-neighbor distances.** Nearest neighbors were computed from all generated to all real training time series, as well as within all training time series. The whiskers show the minimum and maximum distance (L_1 or the Fourier-Wasserstein distance). The minimum nearest-neighbor distance between real and generated time series is away from zero and similar to or larger than the minimum distance within the set of training time series.

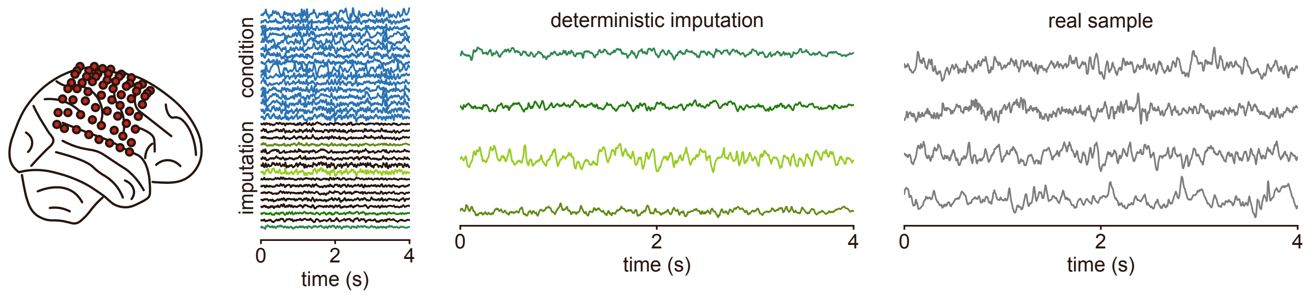

Figure S5: **Example of deterministic neural network-based imputation for the AJILE12 dataset.** The imputation shown is for participant P07. The deterministic imputation appears too flat when compared to the real sample.

Table S1: **Correlations and decoding accuracies for AJILE12 experiment.** Average correlation between imputed and ground truth channels for all test time series across 5 different dropout seeds for diffusion and neural network based imputations. Number in brackets is standard deviation over different dropout seeds. Relative improvement of decoding accuracy over mean imputation in percentage points (pp) when using either DDPM, neural-network or CNNAE imputations[2]. The average and standard deviation over 5 different dropout seeds are shown. First three rows contain the average across all participants.

Pt.	Drop.	Correlation (DDPM)	Correlation (deterministic)	Imp. in pp (DDPM)	Imp. in pp (deterministic)	Imp. in pp Talukder et al.[2]
AVG.	50 %	0.27	0.50	5.9	3.6	3.2
	70 %	0.21	0.45	7.4	6.1	4.6
	90 %	0.10	0.32	4.0	2.5	2.7
P01	50 %	0.37 (0.025)	0.54 (0.026)	−4.6 (5.69)	−10.8 (13.37)	0.2 (0.34)
	70 %	0.28 (0.011)	0.47 (0.013)	0.1 (0.07)	0.0 (0.0)	−0.2 (0.44)
	90 %	0.12 (0.021)	0.32 (0.034)	0.0 (0.04)	0.0 (0.04)	−0.2 (0.39)
P02	50 %	0.42 (0.024)	0.61 (0.020)	2.3 (2.99)	3.1 (2.86)	−1.3 (1.27)
	70 %	0.37 (0.014)	0.58 (0.009)	3.9 (4.55)	5.7 (2.82)	0.5 (2.17)
	90 %	0.18 (0.062)	0.43 (0.049)	4.1 (5.28)	3.9 (2.40)	0.3 (1.08)
P03	50 %	0.30 (0.016)	0.54 (0.015)	8.8 (4.58)	3.9 (1.43)	4.3 (2.66)
	70 %	0.24 (0.006)	0.49 (0.011)	13.7 (3.13)	6.2 (3.69)	4.8 (2.91)
	90 %	0.10 (0.027)	0.32 (0.038)	4.8 (4.07)	0.6 (0.69)	3.6 (4.26)
P04	50 %	0.19 (0.019)	0.38 (0.012)	2.8 (1.88)	6.1 (2.74)	5.0 (2.40)
	70 %	0.15 (0.012)	0.36 (0.022)	4.4 (1.92)	8.8 (1.41)	6.7 (1.81)
	90 %	0.06 (0.012)	0.23 (0.026)	1.0 (1.12)	1.9 (1.93)	1.5 (1.49)
P05	50 %	0.15 (0.007)	0.41 (0.007)	6.8 (2.04)	3.7 (1.02)	2.5 (1.30)
	70 %	0.12 (0.005)	0.37 (0.009)	7.2 (2.24)	3.6 (0.97)	3.1 (0.28)
	90 %	0.05 (0.004)	0.26 (0.011)	2.4 (1.81)	1.2 (0.56)	1.8 (0.88)
P06	50 %	0.11 (0.014)	0.37 (0.020)	0.1 (6.27)	0.6 (3.02)	2.4 (2.37)
	70 %	0.08 (0.009)	0.34 (0.005)	1.8 (3.76)	3.6 (1.22)	1.8 (1.56)
	90 %	0.03 (0.004)	0.23 (0.006)	0.2 (1.11)	−2.2 (3.01)	2.8 (1.33)
P07	50 %	0.35 (0.018)	0.59 (0.015)	14.0 (3.70)	10.6 (3.51)	9.5 (4.77)
	70 %	0.27 (0.025)	0.53 (0.016)	20.7 (1.37)	16.2 (2.68)	13.5 (3.05)
	90 %	0.13 (0.031)	0.38 (0.037)	14.5 (5.23)	7.6 (5.37)	6.7 (1.63)
P08	50 %	0.17 (0.015)	0.43 (0.018)	4.4 (4.37)	2.4 (3.51)	0.6 (2.42)
	70 %	0.13 (0.018)	0.39 (0.021)	2.5 (4.52)	5.7 (1.79)	3.0 (6.00)
	90 %	0.04 (0.007)	0.24 (0.019)	2.8 (1.38)	6.6 (1.03)	1.8 (0.47)
P09	50 %	0.27 (0.021)	0.52 (0.010)	6.5 (2.09)	3.2 (1.51)	−5.7 (1.84)
	70 %	0.21 (0.010)	0.47 (0.010)	5.9 (3.20)	3.5 (1.08)	0.8 (2.01)
	90 %	0.08 (0.012)	0.32 (0.013)	6.7 (2.39)	2.7 (1.92)	1.0 (1.17)
P10	50 %	0.30 (0.011)	0.53 (0.013)	16.1 (15.37)	8.1 (13.57)	10.2 (2.69)
	70 %	0.23 (0.018)	0.47 (0.022)	9.3 (12.89)	3.0 (9.75)	4.7 (5.57)
	90 %	0.14 (0.008)	0.36 (0.004)	1.6 (1.14)	0.5 (0.28)	0.7 (1.31)
P11	50 %	0.28 (0.014)	0.52 (0.016)	4.5 (4.97)	5.2 (5.04)	7.0 (6.97)
	70 %	0.23 (0.018)	0.47 (0.016)	9.8 (5.04)	8.3 (5.09)	11.8 (5.59)
	90 %	0.12 (0.014)	0.34 (0.018)	6.4 (5.45)	5.3 (3.63)	10.1 (7.89)
P12	50 %	0.28 (0.016)	0.55 (0.018)	8.6 (2.68)	8.0 (0.59)	3.5 (1.65)
	70 %	0.22 (0.011)	0.51 (0.011)	10.0 (2.33)	8.6 (1.57)	4.9 (0.84)
	90 %	0.11 (0.019)	0.37 (0.020)	3.6 (1.77)	1.8 (0.47)	1.7 (1.20)

Table S2: **Hyperparameters used to train models.** For the AJILE dataset, he same hyperparameters were used for all 12 participants, with the exception of the number of training epochs, to account for the large differences in training data size between participants

**Hyperparameter**	**LFP (rat)**	**AJILE12**	**ECoG (macaque)**	**EEG BCI**
In kernel size	32	1	32	65
Out kernel size	32	1	32	65
SC layers	3	3	3	3
SC kernel size	32	53	101	65
SC scales	5	4	1	1
Latent dim. per channel	64	16	32	16
Off-diag. size	64	4	4	8
Noise process	White noise	OU ($\rho = 10$)	White noise	White noise
Diffusion steps T	500	50	500	500
Learning rate	0.0001	0.0005	0.0004	0.0004
Weight decay	0.01	0.01	0.01	0.01
Train batch size	32	32	32	32
Epochs	500	250/500/1000	500	1000

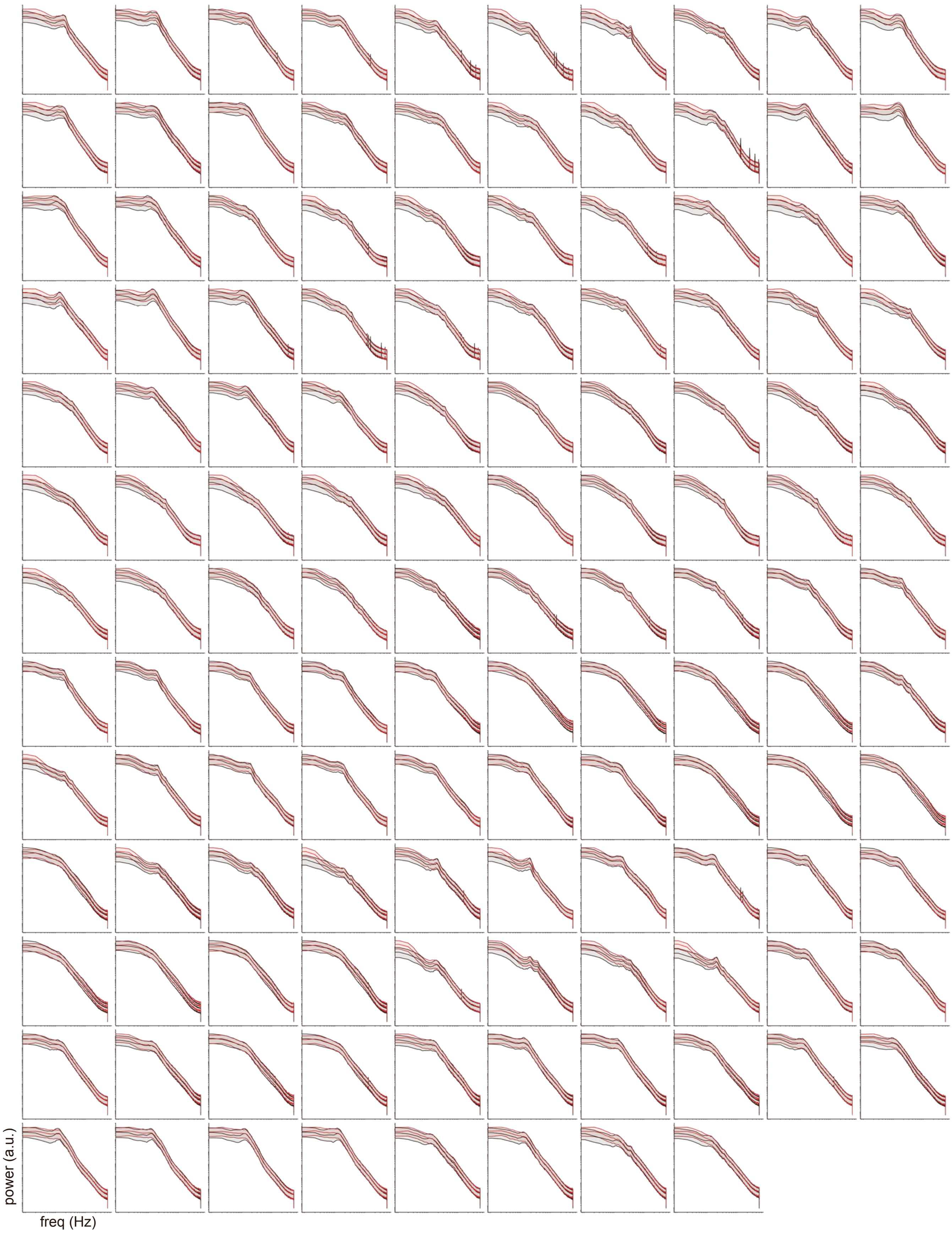

Figure S6: **Full spectra of 128 channel macaque data in awake condition.** Median power as well as 10 %/90 % percentiles are shown.

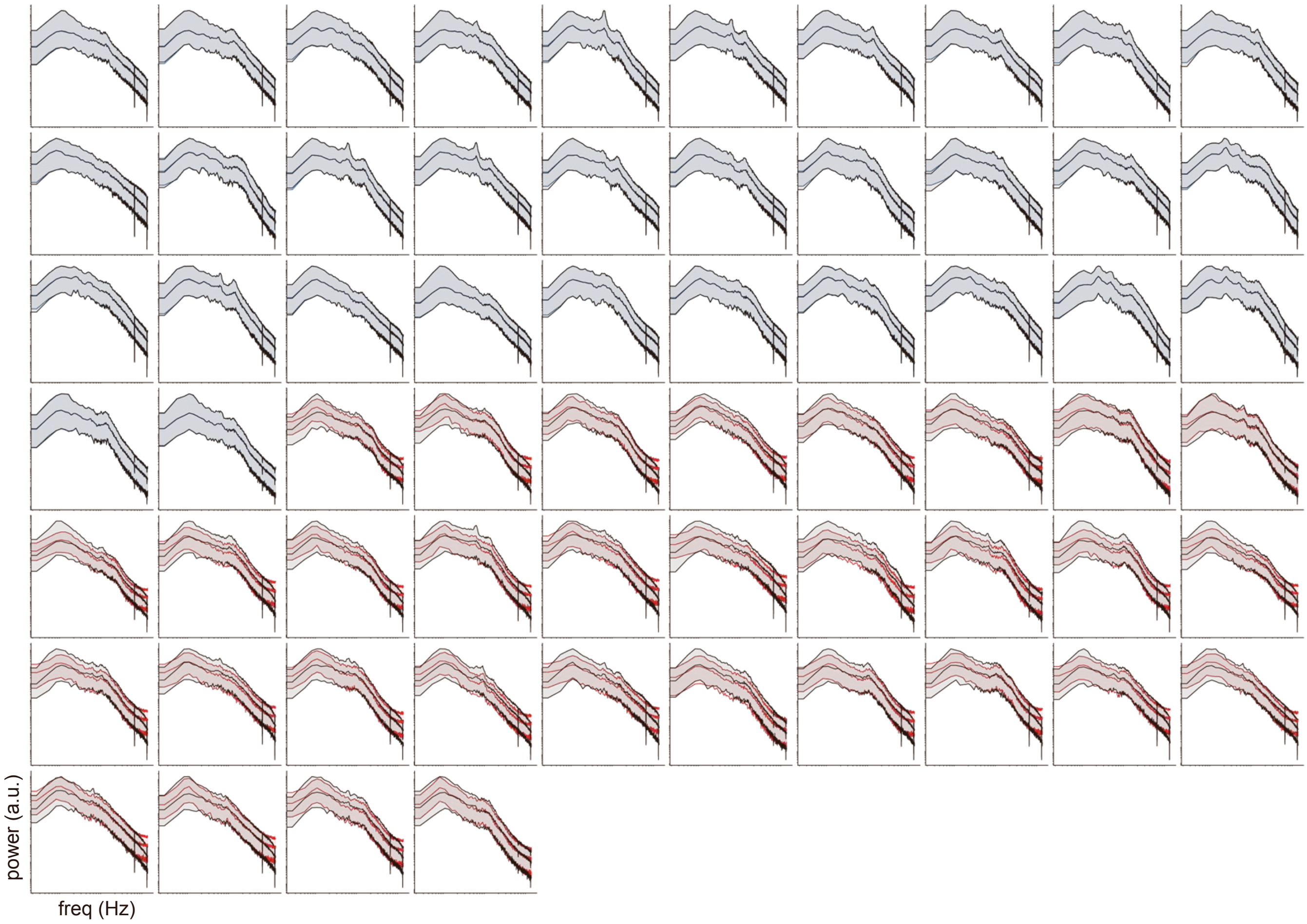

Figure S7: **Full spectra of P07 from the AJILE12 dataset.** The LFP traces consist of 64 channels. Here, we imputed the second half of the channels given the first half for all time series in the evaluation set. Median power as well as 10 %/90 % percentiles are shown.

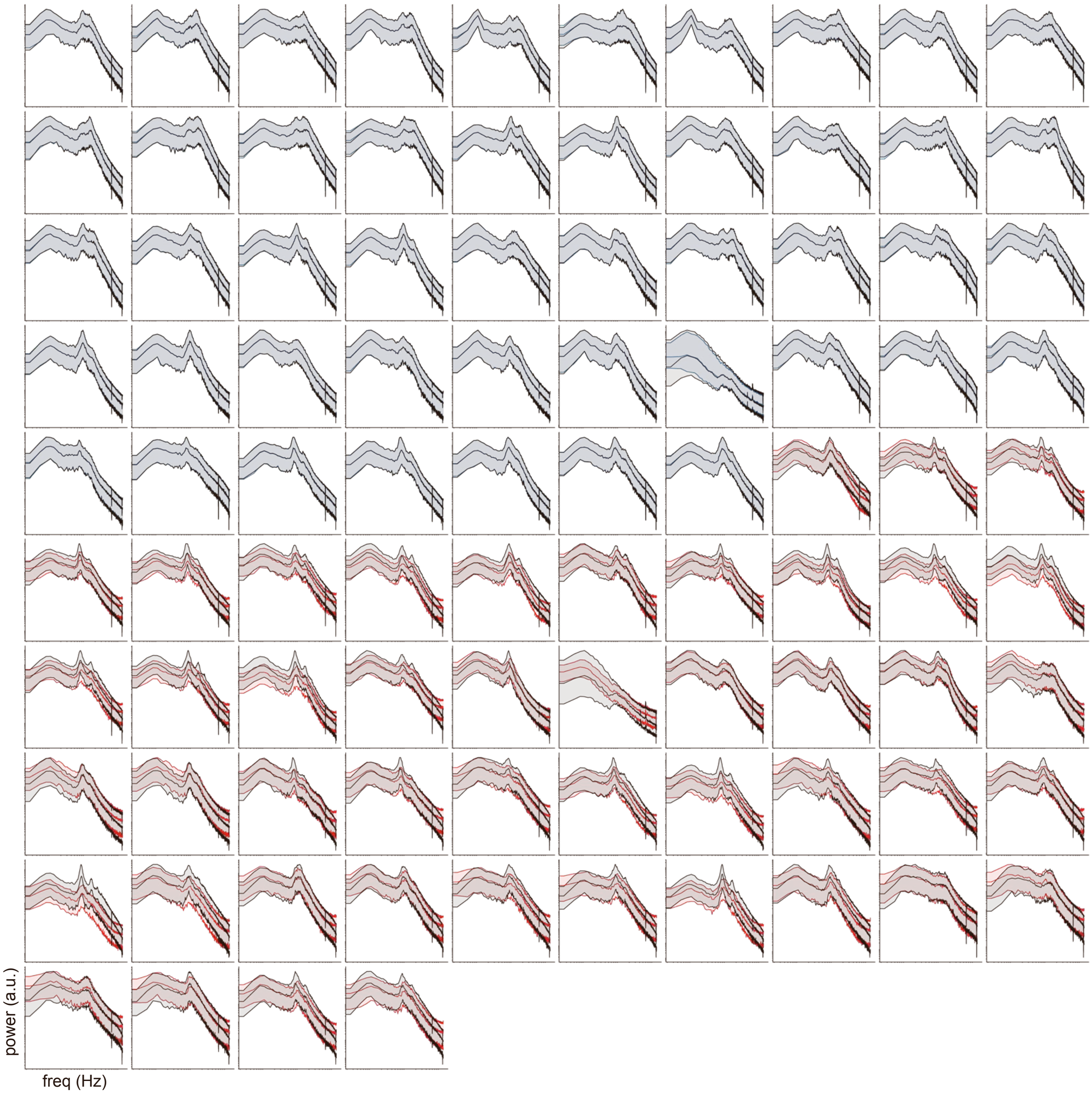

Figure S8: **Full spectra of P01 from the AJILE12 dataset.** The LFP traces consist of 94 channels. Here, we imputed the second half of the channels given the first half for all time series in the evaluation set. Median power as well as 10 %/90 % percentiles are shown.

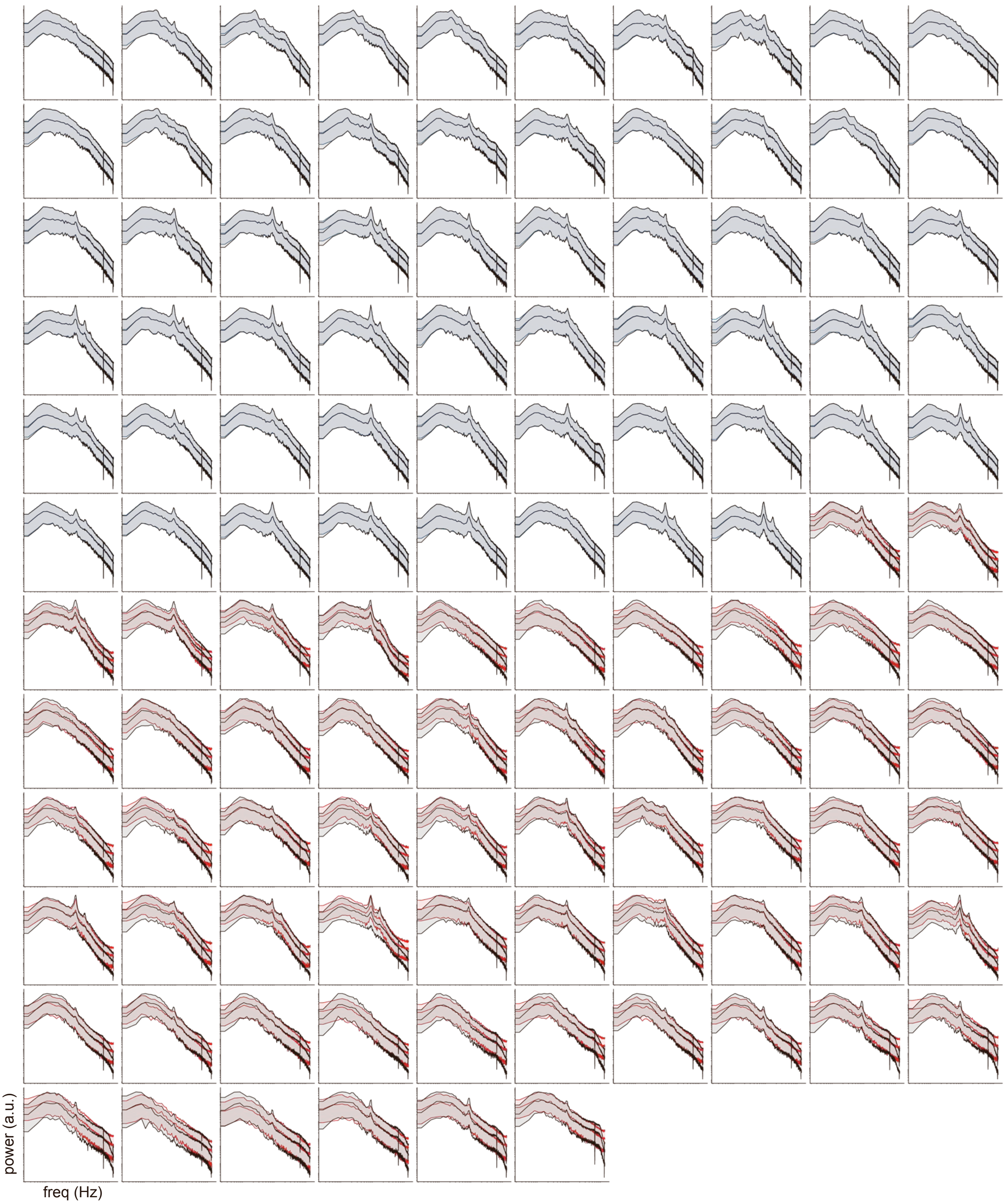

Figure S9: **Full spectra of P12 from the AJILE12 dataset.** The LFP traces consist of 126 channels. Here, we imputed the second half of the channels given the first half for all time series in the evaluation set. Median power as well as 10 %/90 % percentiles are shown.

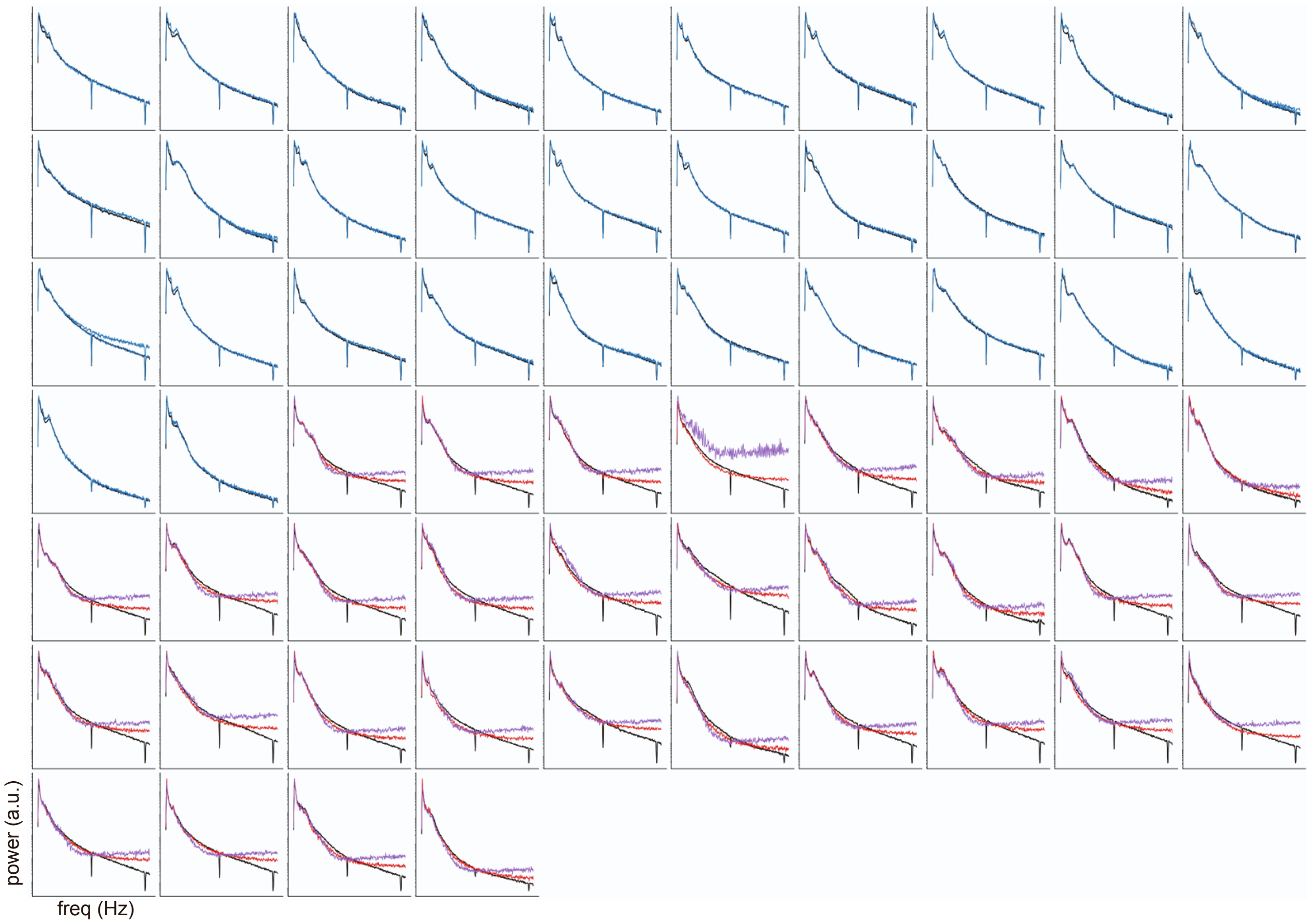

Figure S10: **OU versus white noise diffusion process for P01.** Full (semi-log) median spectra of P01 from the AJILE12 dataset. The second half of channels was imputed given the first half (blue) using a DDPM trained with either white noise (purple) or OU noise (red).

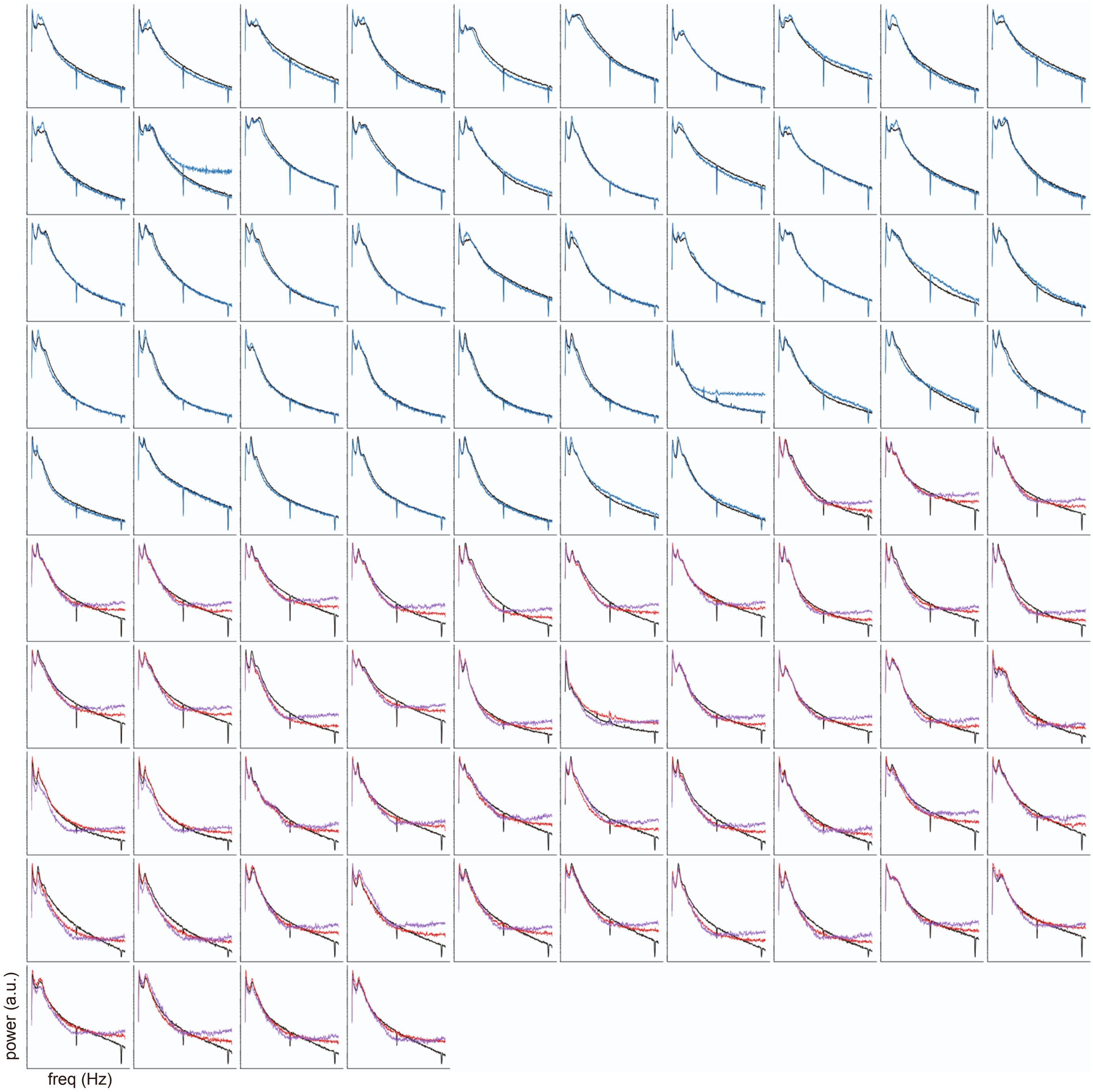

Figure S11: **OU versus white noise diffusion process for P07.** Full (semi-log) median spectra of P07 from the AJILE12 dataset. The second half of channels was imputed given the first half (blue) using a DDPM trained with either white noise (purple) or OU noise (red).

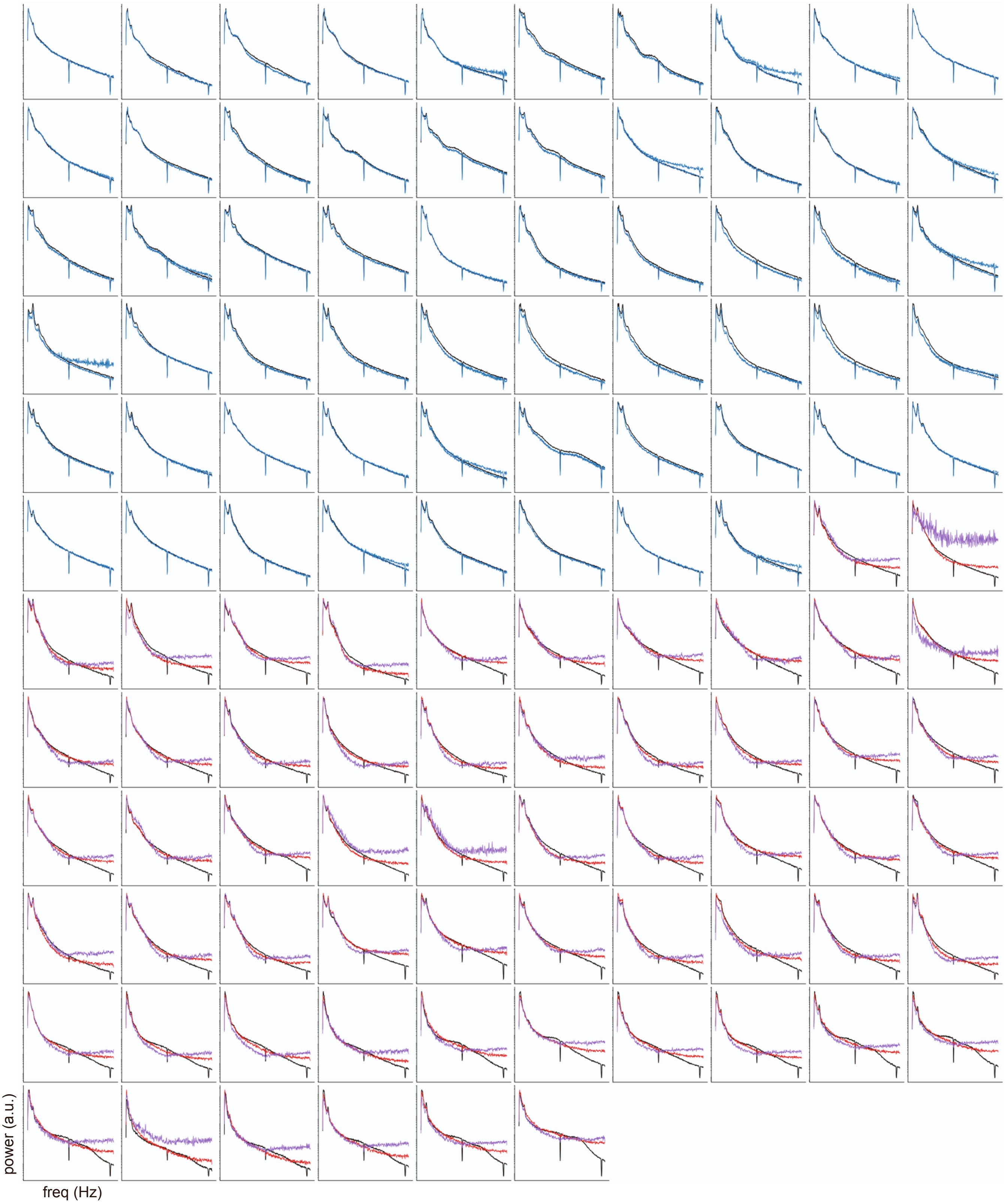

Figure S12: **OU versus white noise diffusion process for P12.** Full (semi-log) median spectra of P12 from the AJILE12 dataset. The second half of channels was imputed given the first half (blue) using a DDPM trained with either white noise (purple) or OU noise (red).

References

1. Biloš, M., Rasul, K., Schneider, A., Nevmyvaka, Y., and Günnemann, S. (2023). Modeling temporal data as continuous functions with process diffusion. International Conference on Machine Learning *202*, 2452–2470.

2. Talukder, S., Sun, J. J., Leonard, M., Brunton, B. W., and Yue, Y. (2022). Deep neural imputation: A framework for recovering incomplete brain recordings. NeurIPS 2022 Workshop on Learning from Time Series for Health.

3. Cazelles, E., Robert, A., and Tobar, F. (2020). The Wasserstein-Fourier distance for stationary time series. IEEE Transactions on Signal Processing *69*, 709–721.

4. Aznan, N. K. N., Atapour-Abarghouei, A., Bonner, S., Connolly, J. D., Al Moubayed, N., and Breckon, T. P. (2019). Simulating brain signals: Creating synthetic eeg data via neural-based generative models for improved ssvep classification. In: 2019 International joint conference on neural networks (IJCNN). (1–8).

5. Kingma, D. P., Welling, M. et al. (2019). An introduction to variational autoencoders. Foundations and Trends in Machine Learning *12*, 307–392.

6. Bredell, G., Flouris, K., Chaitanya, K., Erdil, E., and Konukoglu, E. (2023). Explicitly minimizing the blur error of variational autoencoders. International Conference on Learning Representations *abs/2304.05939*.

7. Kodali, N., Abernethy, J., Hays, J., and Kira, Z. (2017). On convergence and stability of GANs. Preprint at arXiv. https://doi.org/10.48550/arXiv.1705.07215.

8. Mescheder, L., Geiger, A., and Nowozin, S. (2018). Which training methods for GANs do actually converge? International conference on machine learning (3481–3490).
